# Cardiac disease in patients with vasculitis

**DOI:** 10.1007/s00392-025-02728-y

**Published:** 2025-08-25

**Authors:** Leonhard Binzenhöfer, Katharina Strauß, Linus Seifert, Inas Saleh, Marie Scherzer, Julia Höpler, Didzis Gailis, Christina Gebhard, Julia Lichtnekert, Fabian Ullrich, Delila Singh, Torben Sonneck, Matthias Thaler, Sebastian Zimmer, Steffen Massberg, Holger Thiele, Valentin Sebastian Schäfer, Georg Nickenig, Michael Czihal, Hendrik Schulze-Koops, Enzo Lüsebrink

**Affiliations:** 1 Medizinische Klinik Und Poliklinik I, Munich, Germany and DZHK (German Center for Cardiovascular Research), Partner Site Munich Heart Alliance, Klinikum Der Universität München, Munich, Germany; 2 Institute of Medical Information Processing, Biometry and Epidemiology and Department of Statistics, Ludwig-Maximilians Universität München, Munich, Germany; 3 Sektion Rheumatologie Und Klinische Immunologie, Medizinische Klinik Und Poliklinik IV, Klinikum Der Universität München, Pettenkoferstr. 8a, 80336 Munich, Germany; 4 Medizinische Klinik Und Poliklinik II, Universitätsklinikum Bonn, Venusberg-Campus 1, 53127 Bonn, Germany; 5 Department of Internal Medicine/Cardiology and Leipzig Heart Science, Heart Center Leipzig at Leipzig University, Leipzig, Germany; 6 Department of Rheumatology and Clinical Immunology Clinic of Internal Medicine III, University Hospital Bonn, Bonn, Germany; 7 Medizinische Klinik Und Poliklinik IV, Sektion Angiologie, Klinikum Der Universität München, Munich, Germany

**Keywords:** Vasculitis, Structural heart disease, Cardiac arrhythmia

## Abstract

**Background:**

Cardiac involvement has been described in many forms of vasculitides and is associated with worse outcomes. However, data on the incidence of structural and arrhythmic heart disease is limited.

**Methods:**

For this single-center study, we recruited 191 patients with giant-cell arteritis (GCA, *n* = 109), Takayasu arteritis (TAK, *n* = 26), polyarteritis nodosa (PAN, *n* = 3), granulomatosis with polyangiitis (GPA, *n* = 38), or eosinophilic granulomatosis with polyangiitis (EGPA, *n* = 15) between August 2023 and January 2025. The primary study endpoint was the incidence of structural or arrhythmic heart disease after the diagnosis of vasculitis.

**Results:**

The demographic characteristics of patients diagnosed with vasculitis differed significantly between those with GCA, TAK, PAN, GPA, and EGPA. Arterial hypertension and dyslipidemia at baseline were more prevalent among patients with GCA, while chest pain and signs of congestion were more frequently reported by patients with EGPA. No significant difference between the five main subgroups were found regarding the incidence of documented arrhythmic diseases. Cardiac imaging was performed using echocardiography in 70% of the overall cohort and cardiac magnetic resonance (CMR) in 11%. CMR detected left ventricular systolic dysfunction and myocardial fibrosis in 33% and 40% of EGPA patients, respectively. All four cases of acute myocardial infarction occurred in patients with GCA. Among 19 GCA patients who underwent coronary angiography, 21.1% underwent percutaneous coronary intervention. In the EGPA group, coronary angiography was performed in 46.7% of patients, but none required percutaneous intervention. A substantial proportion of patients was treated with acetylsalicylic acid (50.3%), beta-blockers (41.9%), or ACE-inhibitors/AT1-receptor antagonists (60.2%).

**Conclusion:**

Severe cardiac complications occurred rarely, although cardiovascular risk factors, structural abnormalities, and arrhythmias affected a substantial proportion of patients with vasculitis, highlighting the potential benefit of systematic screening and multidisciplinary management.

**Supplementary Information:**

The online version contains supplementary material available at 10.1007/s00392-025-02728-y.

## Introduction

Primary vasculitides are a rare group of systemic autoimmune diseases characterized by inflammation of blood vessel walls and adjacent tissues, and diverse secondary organ damages. The umbrella term primary vasculitis comprises several distinct subtypes categorized according to the predominantly affected vessel size, the histology, and particular serum biomarkers [1, 2]. Cardiac involvement has been described in all major forms of primary vasculitis and is associated with adverse events and increased mortality [3–10]. Cardiac manifestations are heterogeneous, resulting from direct inflammatory involvement or secondary complications, such as aortic valve insufficiency due to aortic root dilation in Takayasu arteritis [11, 12]. Besides, heart disease in patients with primary vasculitis are also related to accelerated atherosclerosis driven by chronic inflammation and may be exacerbated by the administration of glucocorticoids.

Owing to the potential severity of organ manifestations, guidelines for the management of large vessel, medium vessel, and antineutrophil cytoplasmic antibody (ANCA)-associated small vessel vasculitis recommend multidisciplinary care for patients with suspected or confirmed vasculitis [13–17]. In addition, the European Alliance of Associations for Rheumatology (EULAR) has published a clinical practice guideline dedicated to cardiovascular risk management in patients with rheumatic diseases [18]. These include a general recommendation against treatment with platelet inhibitors for primary prevention in vasculitis and underline the importance of remission induction and maintenance as well as glucocorticoid-sparing approaches to decrease cardiovascular risk [18]. However, evidence informing screening algorithms and optimal management of subclinical and manifest cardiac disease in these complex diseases is still sparse.

In this study, the incidence of structural and arrhythmic heart disease was investigated in a large cohort of patients suffering from different primary vasculitides. In addition, data on the prevalence of cardiovascular risk factors, as well as the management of the underlying disease and cardiac comorbidities, was contributed.

## Methods

### Ethics approval

This study was conducted in accordance with the Declaration of Helsinki and approved by the Ludwig-Maximilians-University (LMU) Munich ethics board (IRB-number: 23–0595). Informed consent was obtained from all study participants in written form.

### Study design and patient population

This exploratory study was conducted to investigate the incidence and management of cardiac diseases in patients diagnosed with defined forms of primary vasculitis. The study was conducted between August 2023 and January 2025 at LMU University Hospital in Munich. Adult patients (≥ 18 years) diagnosed with one of the following disease entities were eligible for inclusion in the study: (I) Giant-cell arteritis (GCA), (II) Takayasu arteritis (TAK), (III) Polyarteritis nodosa (PAN), (IV) Granulomatosis with polyangiitis (GPA), or (V) Eosinophilic granulomatosis with polyangiitis (EGPA). The classification of cases followed current guideline recommendations [14, 19–22]. Patients with unconfirmed diagnosis, other vasculitis subtypes, or overlap syndromes were excluded from the study. Patients last seen at our rheumatology or angiology departments before January 2022 were also excluded. The analysis focused on comparisons between the GCA, TAK, GPA and EGPA subgroups. Due to low sample size, the PAN subgroup was not considered for these comparative analyses. Secondary analyses included comparisons between patients with large vessel vasculitis, medium vessel vasculitis, and ANCA-associated small vessel vasculitis as well as comparisons within the large vessel and ANCA-associated vasculitis groups, respectively (see Supplementary appendix).

### Clinical management

The standard of care for patients diagnosed with vasculitis includes quarterly visits to the rheumatology or angiology outpatient clinic for clinical assessment and laboratory testing. If necessary, additional visits, such as for parenteral drug administration, are scheduled on an individual basis. Cardiovascular screening, by means of electrocardiogram, extended laboratory testing, or cardiac imaging, was not routinely performed during these recurrent visits.

### Data acquisition and management

All study participants completed a questionnaire regarding characteristics, disease course and management of vasculitis, as well as cardiovascular risk factors, cardiac symptoms, diagnoses, and management. Information provided by the patients was verified and complemented with the available clinical documentation. Parameters of interest were defined according to current guideline recommendations and clinical consensus statements. Chronic kidney disease was defined as KDIGO stage G3a or worse. Left ventricular (LV) systolic dysfunction was defined as LV ejection fraction < 50% [23]. Right ventricular (RV) dysfunction was defined as tricuspid annular plane systolic excursion (TAPSE) < 18 mm and/or fractional area change ≤ 35% and/or visual impairment of RV contractility. Data validation and assessment of plausibility was performed by an independent study team member. The final dataset was transferred to the Institute of Medical Information Processing, Biometry, and Epidemiology (IBE) at LMU for further analysis and validation.

### Study outcomes

The primary study endpoint was the incidence of structural or arrhythmic heart disease following the diagnosis of vasculitis. Secondary endpoints included cardiac symptoms subsequent to the vasculitis diagnosis, new-onset arterial hypertension, cardiac procedures, and medical management.

### Statistical analysis

All statistical analyses were conducted using the R® software (version 4.4.2, The R Foundation, Vienna, Austria). Continuous variables are presented as medians and interquartile ranges (25th and 75th). Categorical variables are presented as absolute values and percentages. The characteristics of the included patients were compared using the Wilcoxon Rank-Sum test or the Kruskal–Wallis test for continuous variables. Categorical variables were compared using the Fisher's exact test. All tests were conducted with a two-tailed hypothesis, and *p*-values of less than 0.05 were considered statistically significant.

## Results

### Study population

**Fig. 1:**
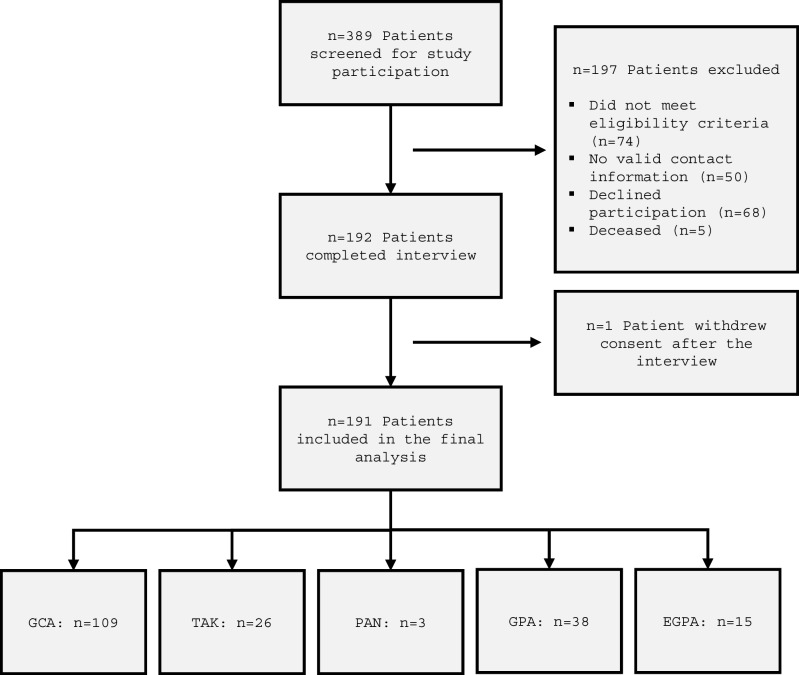
Study flowchart. EGPA, eosinophilic granulomatosis with polyangiitis; GCA, giant cell arteritis; GPA, granulomatosis with polyangiitis; PAN, polyarteritis nodosa; TAK, Takayasu arteritis

In total, 191 patients participated in the study, of whom 109 patients had been diagnosed with GCA, 26 with TAK, three with PAN, 38 with GPA, and 15 with EGPA (Fig. 1). The median age at last follow-up in the overall study population was 72 years. Patients with TAK had the lowest median age (43 years), followed by those with PAN (54 years). In the TAK and GCA subgroups, the majority of patients were female, whereas both GPA and EGPA showed no clear sex preference. Cardiovascular risk factors and comorbidities established before the diagnosis of vasculitis are presented in Table 1. The prevalence of cardiomyopathy, congestive heart failure, and coronary artery disease was each less than 4% in the overall cohort. Overall, 25 patients had undergone invasive coronary angiography, with 20 belonging to the GCA subgroup. Patients with EGPA had significantly more often been diagnosed with a chronic pulmonary disease. In 21.5%, an inflammatory disease other than vasculitis was diagnosed. There were no significant differences regarding the percentage of patients with diabetes, family history of coronary artery disease, or smoking (current or past smoker) between the five main subgroups. By contrast, hypertension and dyslipidemia were seen more frequently in patients with GCA. Further details pertaining to extracardiac organ manifestations and disease-specific parameters can be found in Table S1.

**Table 1:** Demographics, previous medical history, and cardiovascular risk factors

	Overall (*n* = 191)	GCA (*n* = 109) (I)	TAK (*n* = 26) (II)	PAN (*n* = 3) (III)	GPA (*n* = 38) (IV)	EGPA (*n* = 15) (V)	*p*-value (I vs. II vs. IV vs. V)
Demographics							
Age at last follow-up (years), median (IQR)	72.00 [58.00, 79.00]	77.00 [71.00, 83.00]	43.00 [30.25, 49.75]	54.00 [51.00, 69.5]	60.00 [48.00, 72.00]	61.00 [48.50, 68.00]	** < 0.001**
Sex (male), n (%)	68 (35.6)	37 (33.9)	2 (7.7)	1 (33.3)	21 (55.3)	7 (46.7)	**0.001**
Previous medical history							
Cardiomyopathy, n (%)	7 (3.7)	3 (2.8)	2 (7.7)	0 (0.0)	1 (2.6)	1 (6.7)	0.355
Congestive heart failure, n (%)	5 (2.6)	4 (3.7)	0 (0.0)	0 (0.0)	0 (0.0)	1 (6.7)	0.384
Coronary artery disease, n (%)	6 (3.1)	6 (5.5)	0 (0.0)	0 (0.0)	0 (0.0)	0 (0.0)	0.402
Previous myocardial infarction, n (%)	5 (2.6)	5 (4.6)	0 (0.0)	0 (0.0)	0 (0.0)	0 (0.0)	0.552
Previous coronary angiography, n (%)	25 (13.1)	20 (18.3)	1 (3.8)	0 (0.0)	2 (5.3)	2 (13.3)	0.088
Previous percutaneous coronary intervention, n (%)	5 (2.6)	5 (4.6)	0 (0.0)	0 (0.0)	0 (0.0)	0 (0.0)	0.552
Previous vascular intervention other than coronary, n (%)	9 (4.7)	6 (5.5)	2 (7.7)	0 (0.0)	1 (2.6)	0 (0.0)	0.728
Chronic kidney disease, n (%)	11 (5.8)	10 (9.2)	0 (0.0)	0 (0.0)	1 (2.6)	0 (0.0)	0.247
Chronic pulmonary disease, n (%)	27 (14.1)	16 (14.7)	0 (0.0)	1 (33.3)	0 (0.0)	10 (66.7)	** < 0.001**
Chronic inflammatory disease other than vasculitis, n (%)	41 (21.5)	25 (22.9)	9 (34.6)	2 (66.7)	2 (5.3)	3 (20.0)	**0.017**
Cardiovascular risk factors							
Diabetes mellitus, n (%)	29 (15.2)	21 (19.3)	2 (7.7)	0 (0.0)	6 (15.8)	0 (0.0)	0.176
Arterial hypertension, n (%)	77 (40.3)	61 (56.0)	5 (19.2)	0 (0.0)	8 (21.1)	3 (20.0)	** < 0.001**
Dyslipidaemia, n (%)	66 (34.6)	50 (45.9)	3 (11.5)	0 (0.0)	11 (28.9)	2 (13.3)	**0.001**
Smoking history or active smoker, n (%)	82 (42.9)	47 (43.1)	7 (26.9)	1 (33.3)	19 (50.0)	8 (53.3)	0.247
Active smoker at time of diagnosis, n (%)	25 (30.5)	16 (34.0)	4 (57.1)	0 (0.0)	4 (21.1)	1 (12.5)	0.966
Pack years, median (IQR)	12.00 [5.00, 30.00]	15.00 [5.50, 30.00]	10.00 [3.50, 13.75]	-	7.50 [1.00, 27.50]	12.50 [8.62, 45.00]	0.058
Family history of coronary artery disease, n (%)	65 (34.0)	43 (39.4)	7 (26.9)	1 (33.3)	11 (28.9)	3 (20.0)	0.349

*EGPA*, eosinophilic granulomatosis with polyangiitis; *GCA*, giant cell arteritis; *GPA*, granulomatosis with polyangiitis; *IQR*, interquartile range; *PAN*, polyarteritis nodosa; *TAK*, Takayasu arteritis

### Diagnosis of vasculitis

The age at which vasculitis was diagnosed differed significantly across the subgroups (Table 2). GCA was diagnosed at a median of 72 years, whereas the median ages for diagnosis were 31 years for TAK, 44 years for PAN, 51 years for GPA, and 53 years for EGPA. In approximately 80% of patients in the overall population, the respective vasculitis was confirmed within the first year after the initial presentation for disease-related symptoms. This was true for 89.9% of patients with GCA, while it ranged from 60.0% to 71.1% in the other four subgroups. Notably, one in five patients with TAK and EGPA, respectively, received their final diagnosis more than five years after symptom onset. The medical specialties initially suspecting vasculitis are presented in Table 2. GCA was primarily suspected by general practitioners, rheumatologists, ophthalmologists, and in the emergency department. Within the EGPA subgroup, 26.7% of cases were suspected by cardiologists, and 20.0% by pulmonologists.

**Table 2:** Diagnosis of vasculitis

	Overall (*n* = 191)	GCA (*n* = 109) (I)	TAK (*n* = 26) (II)	PAN (*n* = 3) (III)	GPA (*n* = 38) (IV)	EGPA (*n* = 15) (V)	*p*-value (I vs. II vs. IV vs. V)
Diagnosis of vasculitis							
Age at diagnosis (years), median (IQR)	64.00 [48.50, 74.00]	72.00 [66.00, 79.00]	30.50 [22.25, 40.00]	44.00 [37.50, 55.50]	51.00 [38.00, 59.25]	53.00 [44.50, 64.50]	** < 0.001**
Follow-up duration (months), median (IQR)	46.00 [24.00, 115.00]	36.00 [19.00, 65.00]	115.00 [41.00, 157.00]	202.00 [161.00, 210.00]	122.00 [43.50, 199.75]	34.00 [18.50, 93.50]	** < 0.001**
BMI at diagnosis (kg/m^2^), median (IQR)	24.53 [21.35, 27.10]	24.65 [21.90, 26.98]	21.05 [19.87, 26.35]	21.30 [20.32, 21.79]	26.03 [23.48, 28.85]	25.86 [21.98, 30.72]	**0.027**
Number of physician visits until establishment of definite diagnosis (n), median (IQR)	4.00 [2.00, 9.75]	3.00 [2.00, 5.00]	10.00 [5.00, 14.25]	6.00 [4.00, 8.00]	7.00 [3.00, 10.00]	6.00 [3.00, 9.00]	** < 0.001**
Interval between first physician contact related to vasculitis symptoms and confirmed diagnosis							
< = 1 year, n (%)	152 (79.6)	98 (89.9)	16 (61.5)	2 (66.7)	27 (71.1)	9 (60.0)	**0.001**
1 to 2 years, n (%)	12 (6.3)	5 (4.6)	1 (3.8)	1 (33.3)	3 (7.9)	2 (13.3)	0.360
2 to 3 years, n (%)	8 (4.2)	3 (2.8)	2 (7.7)	0 (0.0)	3 (7.9)	0 (0.0)	0.103
3 to 4 years, n (%)	3 (1.6)	0 (0.0)	0 (0.0)	0 (0.0)	2 (5.3)	1 (6.7)	**0.029**
4 to 5 years, n (%)	2 (1.0)	1 (0.9)	0 (0.0)	0 (0.0)	1 (2.6)	0 (0.0)	0.650
> 5 years, n (%)	10 (5.2)	2 (1.8)	5 (19.2)	0 (0.0)	0 (0.0)	3 (20.0)	** < 0.001**
Medical specialty initially suspecting vasculitis							
Rheumatology, n (%)	44 (23.0)	21 (19.3)	10 (38.5)	1 (33.3)	7 (18.4)	5 (33.3)	0.135
Cardiology, n (%)	16 (8.4)	7 (6.4)	5 (19.2)	0 (0.0)	0 (0.0)	4 (26.7)	**0.002**
Angiology, n (%)	17 (8.9)	12 (11.0)	5 (19.2)	0 (0.0)	0 (0.0)	0 (0.0)	**0.021**
Dermatology, n (%)	4 (2.1)	2 (1.8)	0 (0.0)	1 (33.3)	1 (2.6)	0 (0.0)	1.000
Ophthalmology, n (%)	26 (13.6)	21 (19.3)	0 (0.0)	0 (0.0)	4 (10.5)	1 (6.7)	**0.037**
Neurology, n (%)	10 (5.2)	10 (9.2)	0 (0.0)	0 (0.0)	0 (0.0)	0 (0.0)	0.092
Orthopedics, n (%)	6 (3.1)	4 (3.7)	2 (7.7)	0 (0.0)	0 (0.0)	0 (0.0)	0.361
Otorhinolaryngology, n (%)	8 (4.2)	1 (0.9)	0 (0.0)	0 (0.0)	6 (15.8)	1 (6.7)	**0.001**
Pulmonology, n (%)	4 (2.1)	0 (0.0)	0 (0.0)	0 (0.0)	1 (2.6)	3 (20.0)	**0.001**
General medicine, n (%)	48 (25.1)	29 (26.6)	7 (26.9)	2 (66.7)	10 (26.3)	0 (0.0)	0.098
Emergency medicine, n (%)	29 (15.2)	21 (19.3)	0 (0.0)	0 (0.0)	3 (7.9)	5 (33.3)	**0.005**
Other, n (%)	24 (12.6)	10 (9.2)	3 (11.5)	0 (0.0)	10 (26.3)	1 (6.7)	**0.050**

*BMI*, body mass index; *EGPA*, eosinophilic granulomatosis with polyangiitis; *GCA*, giant cell arteritis; *GPA*, granulomatosis with polyangiitis; *IQR*, interquartile range; *PAN*, polyarteritis nodosa; *TAK*, Takayasu arteritis

### Cardiac disease after diagnosis of vasculitis

**Fig. 2:**
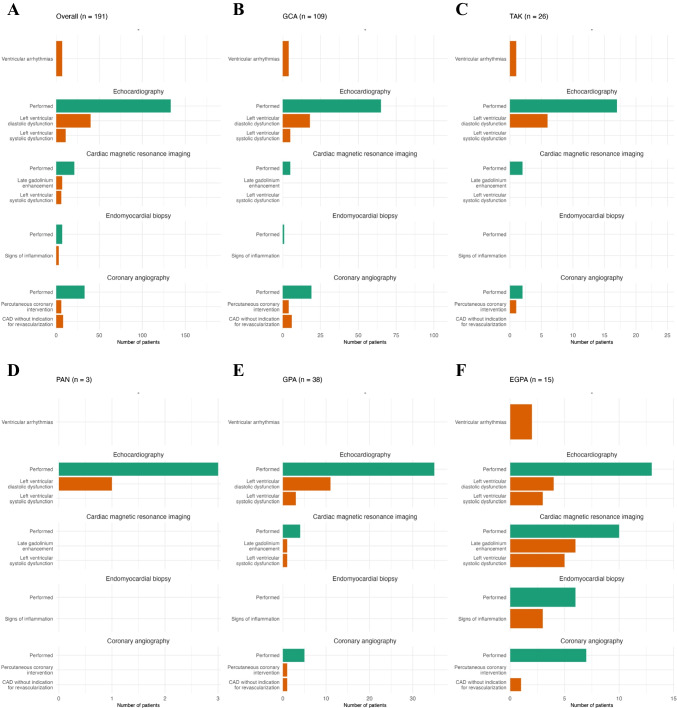
Diagnostic procedures and findings. CAD, coronary artery disease; EGPA, eosinophilic granulomatosis with polyangiitis; GCA, giant cell arteritis; GPA, granulomatosis with polyangiitis; PAN, polyarteritis nodosa; TAK, Takayasu arteritis

Table 3 provides detailed information on new cardiac symptoms, incident hypertension, arrhythmias, and imaging findings including endomyocardial biopsy (EMB) results. Chest pain was reported by 17.8% overall, most frequently in patients with EGPA (46.7%). Clinical signs of congestion were commonly noted after diagnosis of EGPA (53.3%) and PAN (66.7%), but rarely after diagnosis of TAK (7.7%). Arterial hypertension was diagnosed in 27.2% of patients following vasculitis. There were no significant differences across diagnosis groups regarding new supraventricular arrhythmias, ventricular arrhythmias, or conduction abnormalities, which were documented in 11.5%, 3.7%, and 6.3%, respectively. Approximately 70% of study participants underwent basic cardiac imaging with echocardiography (Fig. 2). The most common finding was LV diastolic dysfunction (40/133 patients). In the EGPA subgroup, pericardial effusion was observed in four cases, and LV systolic dysfunction in three cases. Cardiac magnetic resonance imaging (CMR) was performed significantly more often in those with EGPA, showing reduced LV function in 50% and late gadolinium enhancement (LGE) in 60%. EMB was performed almost exclusively in EGPA patients, with histopathologic findings of myocardial inflammation in 50% (three out of six EGPA patients). All four cases of myocardial infarction occurred in patients with GCA.

**Table 3:** Cardiac symptoms and cardiovascular diagnostic findings after vasculitis diagnosis

	Overall (*n* = 191)	GCA (*n* = 109) (I)	TAK (*n* = 26) (II)	PAN (*n* = 3) (III)	GPA (*n *= 38) (IV)	EGPA (*n *= 15) (V)	*p*-value (I vs. II vs. IV vs. V)
**Cardiac symptoms after vasculitis diagnosis**							
Dyspnoea, n (%)	47 (24.6)	24 (22.0)	5 (19.2)	1 (33.3)	10 (26.3)	7 (46.7)	0.222
Chest pain, n (%)	34 (17.8)	13 (11.9)	4 (15.4)	1 (33.3)	9 (23.7)	7 (46.7)	**0.010**
Congestion/oedema, n (%)	50 (26.2)	29 (26.6)	2 (7.7)	2 (66.7)	9 (23.7)	8 (53.3)	**0.014**
Palpitations, n (%)	51 (26.7)	26 (23.9)	9 (34.6)	1 (33.3)	8 (21.1)	7 (46.7)	0.173
Syncope, n (%)	25 (13.1)	18 (16.5)	1 (3.8)	1 (33.3)	3 (7.9)	2 (13.3)	0.269
**New onset arterial hypertension after vasculitis diagnosis**							
New onset arterial hypertension requiring medical therapy after diagnosis of vasculitis, n (%)	52 (27.2)	23 (21.1)	9 (34.6)	1 (33.3)	15 (39.5)	4 (26.7)	0.123
**Arrhythmias after vasculitis diagnosis**							
Conduction disorders, n (%)	12 (6.3)	6 (5.5)	2 (7.7)	0 (0.0)	2 (5.3)	2 (13.3)	0.565
Ventricular arrhythmias, n (%)	7 (3.7)	4 (3.7)	1 (3.8)	0 (0.0)	0 (0.0)	2 (13.3)	0.155
Supraventricular arrhythmias, n (%)	22 (11.5)	16 (14.7)	1 (3.8)	0 (0.0)	3 (7.9)	2 (13.3)	0.438
**New structural abnormalities and biopsy findings after vasculitis diagnosis**							
Echocardiography performed, n (%)	133 (69.6)	65 (59.6)	17 (65.4)	3 (100.0)	35 (92.1)	13 (86.7)	** < 0.001**
Left ventricular systolic dysfunction, n (%)	11 (8.3)	5 (7.7)	0 (0.0)	0 (0.0)	3 (8.6)	3 (23.1)	0.206
Regional wall motion abnormalities, n (%)	4 (3.0)	1 (1.5)	1 (5.9)	0 (0.0)	1 (2.9)	1 (7.7)	0.210
Left ventricular diastolic dysfunction, n (%)	40 (30.1)	18 (27.7)	6 (35.3)	1 (33.3)	11 (31.4)	4 (30.8)	0.827
Right ventricular systolic dysfunction, n (%)	5 (3.8)	3 (4.6)	0 (0.0)	0 (0.0)	2 (5.7)	0 (0.0)	> 0.999
Moderate/severe aortic valve regurgitation, n (%)	4 (3.0)	3 (4.6)	1 (5.9)	0 (0.0)	0 (0.0)	0 (0.0)	> 0.999
Moderate/severe aortic valve stenosis, n (%)	7 (5.3)	3 (4.6)	0 (0.0)	1 (33.3)	3 (8.6)	0 (0.0)	0.626
Moderate severe mitral valve regurgitation, n (%)	12 (9.0)	7 (10.8)	2 (11.8)	1 (33.3)	1 (2.9)	1 (7.7)	0.426
Pericardial effusion, n (%)	12 (9.0)	3 (4.6)	2 (11.8)	0 (0.0)	3 (8.6)	4 (30.8)	**0.042**
Tricuspid regurgitation gradient > 31 mmHg, n (%)	12 (9.0)	7 (10.8)	1 (5.9)	1 (33.3)	1 (2.9)	2 (15.4)	0.364
Cardiac magnetic resonance imaging performed, n (%)	21 (11.0)	5 (4.6)	2 (7.7)	0 (0.0)	4 (10.5)	10 (66.7)	** < 0.001**
Left ventricular systolic dysfunction, n (%)	6 (28.6)	0 (0.0)	0 (0.0)	-	1 (25.0)	5 (50.0)	0.223
Late gadolinium enhancement, n (%)	7 (33.3)	0 (0.0)	0 (0.0)	-	1 (25.0)	6 (60.0)	-
Pericardial effusion, n (%)	2 (9.5)	0 (0.0)	0 (0.0)	-	0 (0.0)	2 (20.0)	-
Endomyocardial biopsy performed, n (%)	7 (3.7)	1 (0.9)	0 (0.0)	0 (0.0)	0 (0.0)	6 (40.0)	** < 0.001**
Signs of inflammation, n (%)	3 (42.9)	0 (0.0)	-	-	-	3 (50.0)	> 0.999
Intracardiac thrombus, n (%)	3 (1.6)	2 (1.8)	0 (0.0)	0 (0.0)	0 (0.0)	1 (6.7)	0.364
**Myocardial ischemia after vasculitis diagnosis**							
Electrocardiographic signs of myocardial ischemia, n (%)	5 (2.6)	3 (2.8)	1 (3.8)	0 (0.0)	0 (0.0)	1 (6.7)	0.420
Myocardial infarction, n (%)	4 (2.1)	4 (3.7)	0 (0.0)	0 (0.0)	0 (0.0)	0 (0.0)	0.726

*EGPA*, eosinophilic granulomatosis with polyangiitis; *GCA*, giant cell arteritis; *GPA*, granulomatosis with polyangiitis; *IQR*, interquartile range; *LV*, left ventricular; *PAN*, polyarteritis nodosa; *TAK*, Takayasu arteritis

### Management of vasculitis

**Fig. 3:**
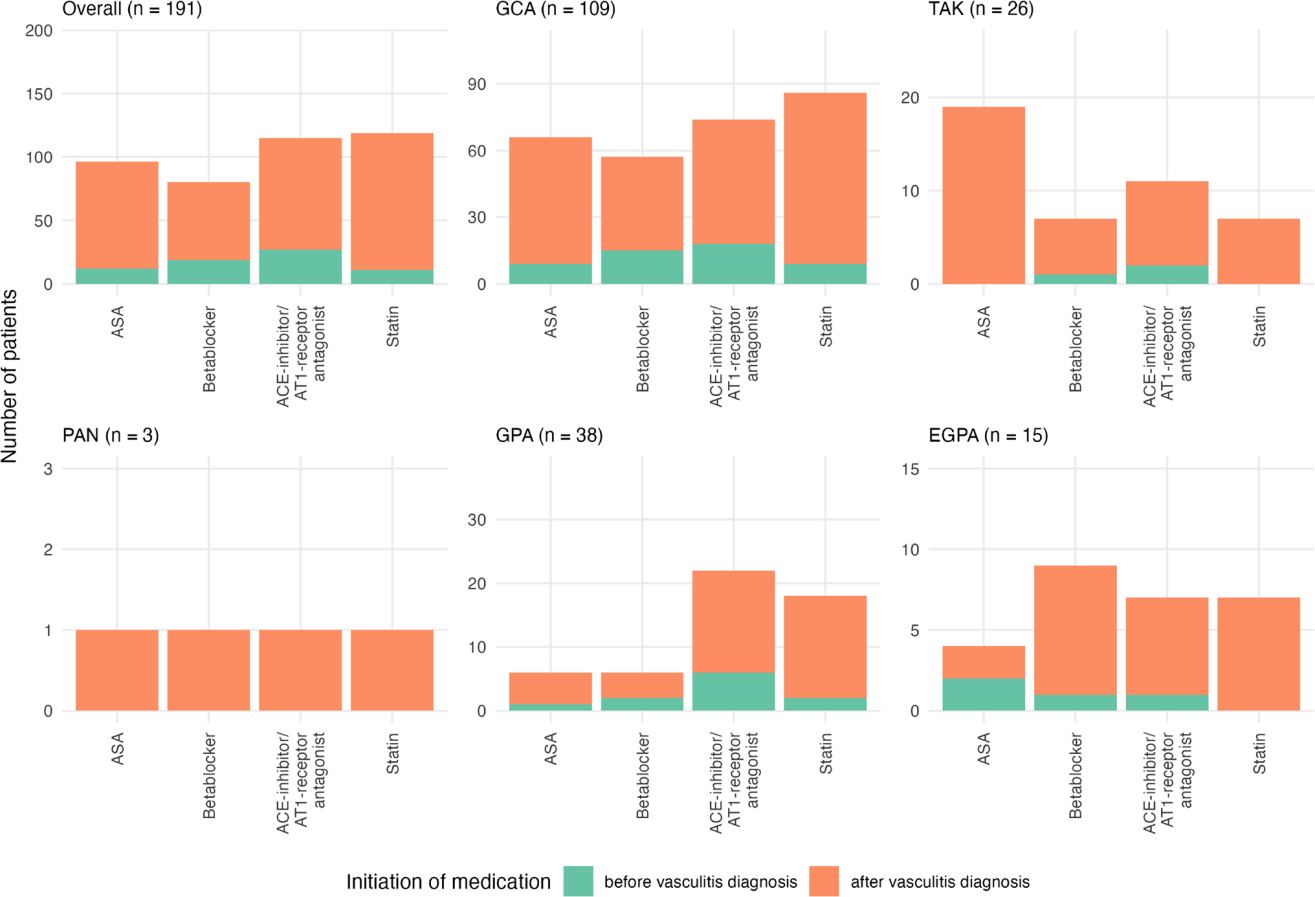
Cardiovascular medication. ACE, angiotensin converting enzyme; ASA, acetyl salicylic acid; AT, angiotensin; EGPA, eosinophilic granulomatosis with polyangiitis; GCA, giant cell arteritis; GPA, granulomatosis with polyangiitis; PAN, polyarteritis nodosa; TAK, Takayasu arteritis

All but two patients received glucocorticoids as initial treatment (Table 4). In addition, 82.7% were administered disease-modifying antirheumatic drugs (DMARDs) or other immunomodulatory medications. Over the disease course, all patients with PAN, GPA, and EGPA were treated with at least one additional agent, but only 70.6% in the GCA subgroup. In the overall cohort, Methotrexate (39.8%), Azathioprine (20.4%), and Cyclophosphamide (11.5%) were the most frequently used immunosuppressants. Besides, commonly used immunomodulators were IL6 receptor antagonists (39.3%), Rituximab (18.3%), TNF antagonists (11.5%), and IL5/IL5 receptor antagonists (6.8%). The use of these additional medications varied significantly between the main subgroups (Table 4). Besides, a substantial proportion of patients was treated with acetylsalicylic acid (ASA) (50.3%), a beta-blocker (41.9%), or an ACE-inhibitor/AT1-receptor antagonist (60.2%) (Fig. 3). Significant differences in prescription rates between subgroups were observed, with ASA being more commonly used by patients with GCA and TAK. Coronary angiography was performed in 33 patients. Among the 19 GCA patients who underwent coronary angiography, 31.6% had coronary artery disease without an indication for revascularization, while 21.1% underwent percutaneous coronary intervention. In the EGPA group, 46.7% underwent invasive coronary angiography, but none required percutaneous intervention. Surgical or interventional valvular procedures, pacemaker implantation, or implantable cardioverter defibrillator (ICD) implantation were performed in individual cases, with no significant differences observed between the groups.

**Table 4:** Management of vasculitis and cardiac disease

	Overall (*n* = 191)	GCA (*n* = 109) (I)	TAK (*n* = 26) (II)	PAN (*n* = 3) (III)	GPA (*n* = 38) (IV)	EGPA (*n* = 15) (V)	*p*-value (I vs. II vs. IV vs. V)
Medical management of vasculitis							
Corticosteroids for initial immunomodulatory therapy, n (%)	189 (99.0)	109 (100.0)	25 (96.2)	3 (100.0)	37 (97.4)	15 (100.0)	0.417
Oral application of corticosteroids, n (%)	161 (85.2)	87 (79.8)	25 (100.0)	3 (100.0)	34 (91.9)	12 (80.0)	0.133
Intravenous application of corticosteroids, n (%)	91 (48.1)	57 (52.3)	6 (24.0)	0 (0.0)	19 (51.4)	9 (60.0)	**0.021**
Long-term immunomodulatory medication before vasculitis diagnosis, n (%)	31 (16.2)	15 (13.8)	5 (19.2)	1 (33.3)	4 (10.5)	6 (40.0)	0.070
Adjunctive immunomodulatory therapy, n (%)	158 (82.7)	77 (70.6)	25 (96.2)	3 (100.0)	38 (100.0)	15 (100.0)	** < 0.001**
Conventional synthetic DMARDs							
Azathioprine, n (%)	39 (20.4)	7 (9.1)	6 (24.0)	2 (66.7)	18 (47.4)	6 (40.0)	** < 0.001**
Methotrexate, n (%)	76 (39.8)	27 (24.8)	20 (76.9)	3 (100.0)	22 (57.9)	4 (26.7)	** < 0.001**
Mycophenolate, n (%)	8 (4.2)	2 (1.8)	0 (0.0)	2 (66.7)	3 (7.9)	1 (6.7)	0.150
Cyclophosphamide, n (%)	22 (11.5)	2 (1.8)	2 (7.7)	2 (66.7)	11 (28.9)	5 (33.3)	** < 0.001**
Hydroxychloroquine, n (%)	2 (1.0)	0 (0.0)	1 (3.8)	0 (0.0)	0 (0.0)	1 (6.7)	**0.047**
Cyclosporin A, n (%)	2 (1.0)	1 (0.9)	1 (3.8)	0 (0.0)	0 (0.0)	0 (0.0)	0.435
Leflunomide, n (%)	8 (4.2)	4 (3.7)	2 (7.7)	0 (0.0)	1 (2.6)	1 (6.7)	0.519
Biologic DMARDs							
IL5 (receptor) antagonists, n (%)	13 (6.8)	1 (0.9)	0 (0.0)	0 (0.0)	0 (0.0)	12 (80.0)	** < 0.001**
if yes, Mepolizumab, n (%)	11 (84.6)	1 (100.0)	0 (0.0)	0 (0.0)	0 (0.0)	10 (83.3)	** < 0.001**
if yes, Benralizumab, n (%)	6 (46.2)	0 (0.0)	0 (0.0)	0 (0.0)	0 (0.0)	6 (50.0)	** < 0.001**
TNF antagonists, n (%)	22 (11.5)	3 (2.8)	17 (65.4)	0 (0.0)	1 (2.6)	1 (6.7)	** < 0.001**
if yes, Etanercept, n (%)	5 (22.7)	3 (100.0)	0 (0.0)	0 (0.0)	1 (100.0)	1 (100.0)	0.625
if yes, Infliximab, n (%)	8 (36.4)	0 (0.0)	7 (41.2)	0 (0.0)	1 (100.0)	0 (0.0)	** < 0.001**
if yes, Adalimumab, n (%)	17 (77.3)	0 (0.0)	16 (94.1)	0 (0.0)	0 (0.0)	1 (100.0)	** < 0.001**
if yes, Certolizumab, n (%)	2 (9.1)	0 (0.0)	2 (11.8)	0 (0.0)	0 (0.0)	0 (0.0)	**0.025**
if yes, Golimumab, n (%)	1 (4.5)	0 (0.0)	1 (5.9)	0 (0.0)	0 (0.0)	0 (0.0)	0.220
IL1-receptor antagonist/Anakinra, n (%)	1 (0.5)	0 (0.0)	0 (0.0)	0 (0.0)	1 (2.6)	0 (0.0)	0.429
Rituximab, n (%)	35 (18.3)	1 (0.9)	1 (3.8)	2 (66.7)	28 (73.7)	3 (20.0)	** < 0.001**
IL6 receptor antagonists, n (%)	75 (39.3)	63 (57.8)	11 (42.3)	0 (0.0)	1 (2.6)	0 (0.0)	** < 0.001**
if yes, Tocilizumab, n (%)	75 (100.0)	63 (100.0)	11 (100.0)	0 (0.0)	1 (100.0)	0 (0.0)	** < 0.001**
if yes, Sarilumab, n (%)	1 (1.3)	1 (1.6)	0 (0.0)	0 (0.0)	0 (0.0)	0 (0.0)	> 0.999
Abatacept, n (%)	0 (0.0)	0 (0.0)	0 (0.0)	0 (0.0)	0 (0.0)	0 (0.0)	-
Targeted specific DMARDs							
JAK-inhibitors, n (%)	1 (0.5)	0 (0.0)	1 (3.8)	0 (0.0)	0 (0.0)	0 (0.0)	0.220
if yes, Baricitinib, n (%)	1 (100.0)	0 (0.0)	1 (100.0)	0 (0.0)	0 (0.0)	0 (0.0)	0.220
if yes, Tofacitinib, n (%)	0 (0.0)	0 (0.0)	0 (0.0)	0 (0.0)	0 (0.0)	0 (0.0)	-
if yes, Upadacitinib, n (%)	0 (0.0)	0 (0.0)	0 (0.0)	0 (0.0)	0 (0.0)	0 (0.0)	-
Other immunomodulatory medications							
Interferon Alpha, n (%)	1 (0.5)	0 (0.0)	0 (0.0)	0 (0.0)	1 (2.6)	0 (0.0)	0.429
Avacopan, n (%)	1 (0.5)	0 (0.0)	0 (0.0)	0 (0.0)	1 (2.6)	0 (0.0)	0.429
Intravenous immune globulin, n (%)	6 (3.1)	0 (0.0)	0 (0.0)	1 (33.3)	3 (7.9)	2 (13.3)	**0.002**
Cardiac procedures							
Coronary angiography performed, n (%)	33 (17.3)	19 (17.4)	2 (7.7)	0 (0.0)	5 (13.2)	7 (46.7)	**0.023**
CAD without indication for revascularization, n (%)	8 (24.2)	6 (31.6)	0 (0.0)	-	1 (20.0)	1 (14.3)	0.632
Percutaneous coronary intervention, n (%)	6 (18.2)	4 (21.1)	1 (50.0)	-	1 (20.0)	0 (0.0)	0.321
Coronary lesions other than atherosclerotic, n (%)	4 (12.1)	2 (10.5)	0 (0.0)	-	2 (40.0)	0 (0.0)	0.321
Coronary artery bypass grafting, n (%)	2 (6.1)	1 (5.3)	0 (0.0)	-	1 (20.0)	0 (0.0)	0.438
Surgical/interventional valvular procedure, n (%)	8 (4.2)	6 (5.5)	1 (3.8)	0 (0.0)	1 (2.6)	0 (0.0)	0.936
Pacemaker implantation after vasculitis diagnosis, n (%)	1 (0.5)	1 (0.9)	0 (0.0)	0 (0.0)	0 (0.0)	0 (0.0)	> 0.999
ICD implantation after vasculitis diagnosis, n (%)	3 (1.6)	2 (1.8)	0 (0.0)	0 (0.0)	0 (0.0)	1 (6.7)	0.361
Cardiovascular medication							
ASA, n (%)	96 (50.3)	66 (60.6)	19 (73.1)	1 (33.3)	6 (15.8)	4 (26.7)	** < 0.001**
Initiated after vasculitis diagnosis, n (%)	84 (87.5)	57 (86.4)	19 (100.0)	1 (100.0)	5 (83.3)	2 (50.0)	** < 0.001**
P2Y12 inhibitor, n (%)	19 (9.9)	11 (10.1)	5 (19.2)	0 (0.0)	2 (5.3)	1 (6.7)	0.348
Initiated after vasculitis diagnosis, n (%)	14 (73.7)	6 (54.4)	5 (100.0)	-	2 (100.0)	1 (100.0)	0.121
Dual antiplatelet therapy, n (%)	12 (6.3)	8 (7.3)	4 (15.4)	0 (0.0)	0 (0.0)	0 (0.0)	0.063
Initiated after vasculitis diagnosis, n (%)	8 (66.7)	4 (50.0)	4 (100.0)	-	-	-	**0.042**
NOAC, n (%)	39 (20.4)	30 (27.5)	1 (3.8)	0 (0.0)	5 (13.2)	3 (20.0)	**0.022**
Initiated after vasculitis diagnosis, n (%)	32 (82.1)	23 (76.7)	1 (100.0)	-	5 (100.0)	3 (100.0)	0.141
Vitamin K Antagonist, n (%)	13 (6.8)	8 (7.3)	1 (3.8)	0 (0.0)	3 (7.9)	1 (6.7)	0.966
Initiated after vasculitis diagnosis, n (%)	7 (53.8)	4 (50.0)	1 (100.0)	-	2 (66.7)	0 (0.0)	0.924
Betablocker, n (%)	80 (41.9)	57 (52.3)	7 (26.9)	1 (33.3)	6 (15.8)	9 (60.0)	** < 0.001**
Initiated after vasculitis diagnosis, n (%)	61 (76.2)	42 (73.7)	6 (85.7)	1 (100.0)	4 (66.7)	8 (88.9)	**0.002**
Diuretics, n (%)	64 (33.5)	41 (37.6)	7 (26.9)	0 (0.0)	9 (23.7)	7 (46.7)	0.254
Initiated after vasculitis diagnosis, n (%)	53 (82.8)	34 (82.9)	6 (85.7)	-	8 (88.9)	5 (71.4)	0.651
Aldosterone antagonist, n (%)	6 (3.1)	3 (2.8)	2 (7.7)	0 (0.0)	0 (0.0)	1 (6.7)	0.175
Initiated after vasculitis diagnosis, n (%)	4 (66.7)	2 (66.7)	1 (50.0)	-	-	1 (100.0)	0.303
ARNI, n (%)	4 (2.1)	2 (1.8)	0 (0.0)	0 (0.0)	0 (0.0)	2 (13.3)	0.074
Initiated after vasculitis diagnosis, n (%)	4 (100.0)	2 (100.0)	-	-	-	2 (100.0)	0.081
SGLT2-inhibitor, n (%)	17 (8.9)	10 (9.2)	1 (3.8)	0 (0.0)	2 (5.3)	4 (26.7)	0.111
Initiated after vasculitis diagnosis, n (%)	14 (82.4)	8 (80.0)	1 (100.0)	-	2 (100.0)	3 (75.0)	0.310
ACE-inhibitor/AT1-receptor antagonist, n (%)	115 (60.2)	74 (67.9)	11 (42.3)	1 (33.3)	22 (57.9)	7 (46.7)	0.059
Initiated after vasculitis diagnosis, n (%)	88 (76.5)	56 (75.7)	9 (81.8)	1 (100.0)	16 (72.7)	6 (85.7)	0.415
Statin, n (%)	119 (62.3)	86 (78.9)	7 (26.9)	1 (33.3)	18 (47.4)	7 (46.7)	** < 0.001**
Initiated after vasculitis diagnosis, n (%)	108 (90.8)	77 (89.5)	7 (100.0)	1 (100.0)	16 (88.9)	7 (100.0)	** < 0.001**
Calcium antagonist, n (%)	50 (26.2)	34 (31.2)	5 (19.2)	0 (0.0)	8 (21.1)	3 (20.0)	0.490
Initiated after vasculitis diagnosis, n (%)	40 (80.0)	26 (76.5)	5 (100.0)	-	8 (100.0)	1 (33.3)	0.468
Antiarrhythmic medication (other than betablocker), n (%)	28 (14.7)	22 (20.2)	1 (3.8)	0 (0.0)	3 (7.9)	2 (13.3)	0.096
Initiated after vasculitis diagnosis, n (%)	21 (75.0)	17 (77.3)	0 (0.0)	-	2 (66.7)	2 (100.0)	0.058

*ACE*, angiotensin converting enzyme; *ARNI*, angiotensin receptor neprilysin inhibitor; *ASA*, acetyl-salicylic acid; *CAD*, coronary artery disease; *DMARD*, disease-modifying anti-rheumatic drug; *EGPA*, eosinophilic granulomatosis with polyangiitis; *GCA*, giant cell arteritis; *GPA*, granulomatosis with polyangiitis; *ICD*, implantable cardioverter defibrillator; *IL*, interleukin; *IQR*, interquartile range; *JAK*, janus kinase; *LV*, left ventricular; *NOAC*, novel oral anticoagulant; *PAN*, polyarteritis nodosa; *SGLT*, sodium dependent glucose co-transporter; *TAK*, Takayasu arteritis; *TNF*, tumor necrosis factor

## Discussion

This exploratory study provides a comprehensive overview of arrhythmias and structural heart disease in a large cohort of patients with vasculitis. The incidence of ventricular arrhythmias, heart failure with reduced ejection fraction, acute myocardial infarction, and cardiomyopathy was low with less than seven percent in the overall population experiencing these potentially life-threatening events after vasculitis diagnosis. Yet, our analysis also demonstrates that cardiovascular risk factors, cardiac symptoms, and less severe structural abnormalities affect a substantial proportion of patients with vasculitis, highlighting the potential to reduce cardiac morbidity through systematic screening and multidisciplinary management.

The differences between the five main subgroups at baseline, i.e., at the time of vasculitis diagnosis, pose a challenge for the interpretation of this study. Some of these differences are directly related to the diagnostic criteria for specific disease subtypes. For example, age below 60 years is considered an absolute requirement for the diagnosis of TAK, while age of ≥ 50 years is a prerequisite of GCA, according to the 2022 American College of Rheumatology (ACR)/EULAR classification criteria [20, 21]. Further, asthma is a typical early clinical feature of EGPA and an important diagnostic criterion, and in accordance with the high rate of chronic pulmonary disease observed at EGPA diagnosis in our study [15, 24]. The high prevalence of arterial hypertension at baseline among patients with GCA may in part be age-related, but may also be an early manifestation of increased arterial wall stiffness due to aortic involvement [25]. Imbalances of non-modifiable and modifiable cardiovascular risk factors at the time of vasculitis diagnosis, knowledge gaps regarding the specific pathomechanisms of cardiac involvement, and the complex interplay between immunomodulatory medications and cardiovascular risk complicate comparisons between these heterogeneous patient subgroups.

A salient finding of the present study was the relatively high rate of LV systolic dysfunction and myocardial fibrosis after EGPA diagnosis compared to other vasculitis subtypes. The risk of cardiac involvement is known to be more pronounced in ANCA-negative EGPA patients [26–30]. In a French multicenter analysis of 383 EGPA patients, 16.4% had cardiomyopathy at the time of diagnosis [26]. In another prospective study involving 50 EGPA patients, comprehensive cardiologic screening using clinical assessment, laboratory analysis, electrocardiogram, echocardiography, and CMR revealed cardiac involvement in 66% of patients [7]. Importantly, EGPA patients with cardiac involvement had a higher risk of death during the mean follow-up period of 53 months [7]. Other analyses have demonstrated that cardiac involvement commonly affects asymptomatic patients [29]. The observational design of our study did not allow for the detection of subclinical cardiac disease but aligns with previous findings highlighting the high prevalence of cardiac morbidity in EGPA patients [7, 26–33]. Thus, our data also supports the call for incorporating cardiologic screening tests including imaging into the routine diagnostic workup at EGPA diagnosis [15]. Whether or not CMR should be part of a standard screening algorithm remains a matter of debate. In a recent study focusing on CMR findings, the sensitivity of elevated N-terminal pro-B-type natriuretic peptide (NT-proBNP) and Troponin T for cardiac involvement, i.e., focal or multifocal subendocardial LGE, was 38% and 56%, respectively [32]. Evidence regarding the value of repeated imaging and EMB is also scarce. Additional information from longitudinal studies including a detailed analysis of inflammatory disease markers at the time of overt and subclinical cardiac involvement is needed to better understand the pathophysiology and chronicity of structural changes, and to guide clinical management.

Stenotic coronary artery disease was excluded in all seven EGPA patients enrolled in this study who underwent coronary angiography. By contrast, acute myocardial infarction requiring PCI was documented in four GCA patients corresponding to a prevalence of 3.7% over a median follow-up period of 36 months. Although GCA patients appeared to have an increased risk of major adverse cardiovascular events due to the presence of baseline risk factors in our cohort, systemic inflammation, coronary vasculitis, and side effects of immunomodulatory medications may have contributed to myocardial ischemia, as well. Two independent healthcare database analyses conducted in Canada and the UK found a significantly higher incidence rate of myocardial infarction and stroke among GCA patients compared to non-GCA controls after matching for age and sex [9, 34]. In a study by Greigert et al., the incidence of myocardial infarction in a cohort of 251 biopsy-proven GCA patients was 5.2% [35]. Of the 13 total events, the authors classified six cases of myocardial infarction as GCA-related, defined by their occurrence within three months before or after a GCA flare [35]. Interestingly, patients who experienced GCA-unrelated myocardial infarction more often had extensive coronary artery disease and type I myocardial infarction, and tended to have received antiplatelet therapy more frequently prior to the event [35]. In the present cohort, coronary lesions other than atherosclerotic were detected in two patients with GCA and GPA, respectively. Few case reports have described coronary arteritis as a rare complication of GCA, whereas it is more frequently observed in TAK, with coronary involvement reported in up to 53% of cases [10, 36–41]. In GPA, coronary artery aneurysm and dissection have been described in rare cases [42–44]. These observations underscore the complexity of coronary artery involvement in vasculitis and the challenge of reliably estimating the risk of major adverse events in clinical practice. In fact, the 2022 EULAR recommendations for cardiovascular risk management in rheumatic diseases acknowledge, that disease-specific factors may impair the accuracy of traditional risk prediction tools [18]. However, due to the lack of validated alternative models, these established tools remain recommended for vasculitis patients, except in ANCA-associated vasculitis, where the European Vasculitis Society (EUVAS) model should be considered [18].

Inflammatory disease activity has been demonstrated to correlate with cardiovascular risk in ANCA-associated vasculitis [45, 46]. In GCA and TAK, the use of adjunctive immunosuppressive/immunomodulatory medication has been shown to be associated with a reduced rate of cardiovascular events, including stroke and myocardial infarction [47, 48]. Apart from the GCA subgroup, almost all study participants received disease-modifying anti-rheumatic drugs over the disease course. This finding reflects general efforts to implement corticosteroid-sparing agents for vasculitis management but may also be influenced by the recruitment of patients for clinical trials at our university-affiliated tertiary center. Since laboratory markers were not collected in this study, adjudicating major cardiovascular events based on disease activity was not feasible. Thus, we can only speculate that individualized medication management could have contributed to the overall low event rate and attenuated the risks associated with long-term corticosteroid use. A notable result from our analysis of cardiovascular medications was the high proportion of GCA and TAK patients receiving or having previously received platelet inhibitors after a vasculitis diagnosis, possibly because their use for primary prevention in large vessel vasculitis was recommended until 2020 [13, 18, 49]. Besides, patients with LV systolic dysfunction should ideally be treated with a combination of four heart failure medications, if tolerated [23]. However, not all patients with reduced LV ejection fraction had been prescribed an aldosterone antagonist or an angiotensin receptor-neprilysin inhibitor. These findings highlight the importance of multidisciplinary management in preventing overtreatment and optimizing long-term medication plans.

Owing to the variety of initial symptoms and organ manifestations, vasculitis was often diagnosed months -or even years- after symptom onset in the present cohort. In a previous survey of 456 vasculitis patients, 73% were initially misdiagnosed, with a median time of seven months between the onset of symptoms and diagnosis [50]. The range of clinical specialists involved in the diagnosis reflects these challenges and underscores the importance of cardiologists considering these rare diseases, particularly in younger patients with atypical cardiac conditions and suggestive inflammatory comorbidities [51].

### Limitations

Main limitations of the present analysis are related to the retrospective study design and the potential biases associated with interview-based data acquisition. Self-reported data may be subject to recency and recall bias, potentially affecting the accuracy of symptom onset, the time of diagnosis, medication history et cetera. However, this information was double-checked on the basis of clinical records. Since deceased patients were excluded by study design, those with severe cardiac manifestations or comorbidities may have been underrepresented. Given the very small number of patients with PAN, the estimated incidence of cardiac conditions may be unreliable and regression analyses were not feasible due to marked group size imbalances. The timing of cardiovascular events in relation to vasculitis diagnosis was not analyzed, which may have introduced additional bias due to variability in follow-up durations. Lastly, as patients were recruited from a major university hospital with specialized rheumatology and angiology departments, the findings may not be generalizable to other settings.

## Conclusion

In this large cohort of patients suffering from vasculitis, severe cardiac complications were rare. However, despite the widespread use of advanced immunosuppressive and immunomodulatory treatments, a substantial proportion of patients still developed arterial hypertension, or were affected by structural cardiac abnormalities and arrhythmias. These findings highlight the need for a multidisciplinary approach to long-term management, including optimization of common risk factors, and standardized disease-specific screening for cardiac manifestations and comorbidities.

## Supplementary Information

Below is the link to the electronic supplementary material.

Supplementary file1 (DOCX 88 KB)

## Declarations

### Data sharing agreement

The data are not publicly available due to ethical restrictions and legal constraints. Readers may contact the corresponding authors for reasonable requests for the data. De-identified data may be provided after approval from the ethical review board.
