# Supplementary material for: Cardiac disease in patients with vasculitis
Source: Clin Res Cardiol. 2025 Aug 25;114(9):1216–31. doi: 10.1007/s00392-025-02728-y (PMC12408678; doi:10.1007/s00392-025-02728-y)
Supplement: Supplementary file 1 — Supplementary file1 (DOCX 88 KB) [file 392_2025_2728_MOESM1_ESM.docx]

**- Supplementary Materials -**

**Cardiac disease in patients with vasculitis**

Leonhard Binzenhöfer^1*^ MD, Katharina Strauss^1*^ MD, Linus Seifert^1^ MDS, Inas Saleh^1^ MD, Marie Scherzer^2^ BSc, Julia Höpler^2^ BSc, Didzis Gailis^3^ MD, Christina Gebhard^3^ MD, Julia Lichtnekert^3^ MD, Fabian Ullrich^3^ MD, Delila Singh^3^ MD, Torben Sonneck^3^ MD, Matthias Thaler^3^ MD, Sebastian Zimmer^4^ MD, Steffen Massberg^1^ MD, Holger Thiele^5^ MD, Valentin Sebastian Schäfer^6^ MD, Georg Nickenig^4^ MD, Michael Czihal^7^ MD, Hendrik Schulze-Koops^3†^ MD, PhD, Enzo Lüsebrink^4†^ MD

^1^ Medizinische Klinik und Poliklinik I, Klinikum der Universität München, München, Germany and
 DZHK (German Center for Cardiovascular Research), partner site Munich Heart Alliance, München, Germany

^2^ Institute of Medical Information Processing, Biometry and Epidemiology and Department of
Statistics, Ludwig-Maximilians Universität München, München, Germany

^3^ Sektion Rheumatologie und Klinische Immunologie, Medizinische Klinik und Poliklinik IV, Klinikum der Universität München, München, Germany

^4^ Medizinische Klinik und Poliklinik II, Universitätsklinikum Bonn, Bonn, Germany

^5^ Heart Center Leipzig at Leipzig University, Department of Internal Medicine/Cardiology
and Leipzig Heart Science, Leipzig, Germany

^6^ Department of Rheumatology and Clinical Immunology Clinic of Internal Medicine III, University Hospital Bonn, Bonn, Germany.

^7^ Sektion Angiologie, Medizinische Klinik und Poliklinik IV, Klinikum der Universität München, München, Germany

**^*^** **These authors contributed equally to the manuscript as first authors.
^†^** **These authors contributed equally to the manuscript as senior authors.**

|  | | **Overall (n=228)** | **GCA (n=109)**  **(I)** | **TAK (n=26)**  **(II)** | **PAN (n=3)**  **(III)** | **GPA (n=38)**  **(IV)** | **EGPA (n=15)**  **(V)** | **p-value (I vs. II vs. IV vs. V)** |
| --- | --- | --- | --- | --- | --- | --- | --- | --- |
| Hospital admission for vasculitis treatment, n (%) | | 96 (50.3) | 54 (49.5) | 13 (50.0) | 2 (66.7) | 16 (42.1) | 11 (73.3) | 0.302 |
| Major relapse, n (%) | | 63 (33.0) | 29 (26.6) | 9 (34.6) | 2 (66.7) | 19 (50.0) | 4 (26.7) | 0.066 |
| **Extracardiac organ manifestations** | | | | | | | | |
| Stroke/transitory ischemic attack, n (%) | | 11 (5.8) | 7 (6.4) | 1 (3.8) | 1 (33.3) | 2 (5.3) | 0 (0.0) | 0.953 |
| Permanent disability requiring constant nursing care, n (%) | | 9 (4.7) | 7 (6.4) | 1 (3.8) | 1 (33.3) | 0 (0.0) | 0 (0.0) | 0.442 |
| End-stage kidney disease requiring dialysis/kidney transplantation, n (%) | | 1 (0.5) | 0 (0.0) | 0 (0.0) | 0 (0.0) | 1 (2.6) | 0 (0.0) | 0.420 |
| Arterial occlusion requiring intervention/critical limb ischemia/amputation, n (%) | | 6 (3.1) | 3 (2.8) | 1 (3.8) | 0 (0.0) | 2 (5.3) | 0 (0.0) | 0.755 |
| **B-symptoms at systemic vasculitis diagnosis** | | | | | | | | |
| Fever, n (%) | | 51 (26.7) | 28 (25.7) | 5 (19.2) | 1 (33.3) | 14 (36.8) | 3 (20.0) | 0.343 |
| Arthralgias, n (%) | | 102 (53.4) | 58 (53.2) | 10 (38.5) | 1 (33.3) | 24 (63.2) | 9 (60.0) | 0.260 |
| Sicca, n (%) | | 57 (29.8) | 37 (33.9) | 3 (11.5) | 1 (33.3) | 11 (28.9) | 5 (33.3) | 0.198 |
| Night sweats, n (%) | | 91 (47.6) | 57 (52.3) | 7 (26.9) | 1 (33.3) | 21 (55.3) | 5 (33.3) | **0.042** |
| Myalgia, n (%) | | 106 (55.5) | 62 (56.9) | 12 (46.2) | 2 (66.7) | 19 (50.0) | 11 (73.3) | 0.427 |
| Raynaud, n (%) | | 27 (14.1) | 14 (12.8) | 4 (15.4) | 1 (33.3) | 6 (15.8) | 2 (13.3) | 0.930 |
| Weight Loss, n (%) | | 74 (38.7) | 43 (39.4) | 9 (34.6) | 1 (33.3) | 15 (39.5) | 6 (40.0) | 0.984 |
| Hair Loss, n (%) | | 31 (16.2) | 19 (17.4) | 5 (19.2) | 1 (33.3) | 3 (7.9) | 3 (20.0) | 0.491 |
| Bleeding tendency (excluding drug-related), n (%) | | 22 (11.5) | 12 (11.0) | 1 (3.8) | 1 (33.3) | 7 (18.4) | 1 (6.7) | 0.353 |
| **Specific disease characteristics related to GCA** | | | | | | | | |
| Headache, n (%) | | - | 85 (78.0) | - | - | - | - | - |
| Jaw claudication, n (%) | | - | 69 (63.3) | - | - | - | - | - |
| Ocular involvement, n (%) | | - | 54 (49.5) | - | - | - | - | - |
| Transient vision loss, n (%) | | - | 29 (53.7) | - | - | - | - | - |
| Permanent vision loss, n (%) | | - | 30 (55.6) | - | - | - | - | - |
| Cerebral ischemia, n (%) | | - | 3 (2.8) | - | - | - | - | - |
| Central nervous involvement, n (%) | | - | 3 (2.8) | - | - | - | - | - |
| Cerebrovascular disease, n (%) | | - | 8 (7.3) | - | - | - | - | - |
| Peripheral nervous system involvement, n (%) | | - | 7 (6.4) | - | - | - | - | - |
| Peripheral artery disease, n (%) | | - | 7 (6.4) | - | - | - | - | - |
| Musculoskeletal involvement, n (%) | | - | 45 (41.3) | - | - | - | - | - |
| Polymyalgia rheumatica, n (%) | | - | 29 (26.6) | - | - | - | - | - |
| Aortic aneurysm, n (%) | | - | 15 (13.8) | - | - | - | - | - |
| Aortic dissection, n (%) | | - | 2 (1.8) | - | - | - | - | - |
| Upper respiratory tract involvement, n (%) | | - | 13 (11.9) | - | - | - | - | - |
| Cutaneous involvement, n (%) | | - | 10 (9.2) | - | - | - | - | - |
| Infections, n (%) | | - | 13 (11.9) | - | - | - | - | - |
| Malignancy, n (%) | | - | 17 (15.6) | - | - | - | - | - |
| **Specific disease characteristics related to TAK** | | | | | | | | |
| Angiographic classification at diagnosis | Numano 1, n (%) | - | - | 4 (15.4) | - | - | - | - |
|  | Numano 2A, n (%) | - | - | 4 (15.4) | - | - | - | - |
|  | Numano 2B, n (%) | - | - | 3 (11.5) | - | - | - | - |
|  | Numano 3, n (%) | - | - | 2 (7.7) | - | - | - | - |
|  | Numano 4, n (%) | - | - | 0 (0.0) | - | - | - | - |
|  | Numano 5, n (%) | - | - | 6 (23.1) | - | - | - | - |
| Renal artery involvement, n (%) | | - | - | 4 (15.4) | - | - | - | - |
| Thoracic artery involvement, n (%) | | - | - | 15 (57.7) | - | - | - | - |
| Abdominal aorta involvement, n (%) | | - | - | 7 (26.9) | - | - | - | - |
| Pulmonary artery involvement, n (%) | | - | - | 3 (11.5) | - | - | - | - |
| Presence of anti-phospholipid antibodies, n (%) | | - | - | 1 (3.8) | - | - | - | - |
| Acute clinical course, n (%) | | - | - | 13 (50.0) | - | - | - | - |
| **Specific disease characteristics related to PAN** | | | | | | | | |
| Idiopathic generalized disease, n (%) | | - | - | - | 1 (33.3) | - | - | - |
| Hepatitis-B-associated PAN, n (%) | | - | - | - | 0 (0.0) | - | - | - |
| Cutaneous PAN, n (%) | | - | - | - | 2 (66.7) | - | - | - |
| Renal artery involvement, n (%) | | - | - | - | 0 (0.0) | - | - | - |
| Peripheral nervous system involvement, n (%) | | - | - | - | 3 (100.0) | - | - | - |
| Gastrointestinal involvement, n (%) | | - | - | - | 1 (33.3) | - | - | - |
| Renal involvement, n (%) | | - | - | - | 0 (0.0) | - | - | - |
| Antiviral therapy, n (%) | | - | - | - | 1 (33.3) | - | - | - |
| **Specific disease characteristics related to GPA** | | | | | | | | |
| Organ/life threatening manifestation, n (%) | | - | - | - | - | 11 (28.9) | - | - |
| Glomerulonephritis, n (%) | | - | - | - | - | 11 (100.0) | - | - |
| Pulmonary hemorrhage/lung cavitary lesions, n (%) | | - | - | - | - | 7 (63.6) | - | - |
| Meningeal involvement, n (%) | | - | - | - | - | 1 (2.6) | - | - |
| Central nervous system involvement, n (%) | | - | - | - | - | 2 (5.3) | - | - |
| Retro-orbital disease, n (%) | | - | - | - | - | 4 (10.5) | - | - |
| Mesenteric involvement, n (%) | | - | - | - | - | 1 (2.6) | - | - |
| Mononeuritis multiplex, n (%) | | - | - | - | - | 4 (10.5) | - | - |
| Active GPA at admission, n (%) | | - | - | - | - | 18 (47.4) | - | - |
| Malignancy, n (%) | | - | - | - | - | 5 (13.2) | - | - |
| Subglottic stenosis, n (%) | | - | - | - | - | 1 (2.6) | - | - |
| Hypogammaglobulinemia, n (%) | | - | - | - | - | 5 (13.2) | - | - |
| Peripheral nervous system involvement, n (%) | | - | - | - | - | 10 (26.3) | - | - |
| Sinunasal involvement, n (%) | | - | - | - | - | 30 (78.9) | - | - |
| Cutaneous involvement, n (%) | | - | - | - | - | 11 (28.9) | - | - |
| **Specific disease characteristics related to EGPA** | | | | | | | | |
| Plasma exchange therapy performed, n (%) | | - | - | - | - | - | 0 (0.0) | - |
| Organ/life threatening manifestation, n (%) | | - | - | - | - | - | 9 (60.0) | - |
| Renal dysfunction, n (%) | | - | - | - | - | - | 3 (20.0) | - |
| Respiratory involvement, n (%) | | - | - | - | - | - | 3 (20.0) | - |
| Status asthmaticus, n (%) | | - | - | - | - | - | 10 (66.7) | - |
| Central nervous system involvement, n (%) | | - | - | - | - | - | 1 (6.7) | - |
| Gastrointestinal involvement, n (%) | | - | - | - | - | - | 2 (13.3) | - |
| Cutaneous involvement, n (%) | | - | - | - | - | - | 4 (26.7) | - |
| Sinunasal involvement, n (%) | | - | - | - | - | - | 10 (66.7) | - |
| Malignancy, n (%) | | - | - | - | - | - | 0 (0.0) | - |

**Table S1**: Extracardiac organ manifestations, specific symptoms, and disease characteristics related to vasculitis.

|  | **Overall**  **(n=191)** | **LVV**  **(n=135)**  **(I)** | **MVV**  **(n=3)**  **(II)** | **SVV**  **(n=53)**  **(III)** | **p-value**  **I vs. II vs. III** |
| --- | --- | --- | --- | --- | --- |
| **Demographics** | | | | | |
| Age at last follow-up (years), median (IQR) | 72.00 [58.00, 79.00] | 74.00 [65.00, 81.00] | 54.00 [51.00, 69.50] | 60.50 [47.75, 71.25] | **<0.001** |
| Sex (male), n (%) | 68 (35.6) | 39 (28.9) | 1 (33.3) | 28 (52.8) | **0.005** |
| **Previous medical history** | | | | | |
| Cardiomyopathy, n (%) | 7 (3.7) | 5 (3.7) | 0 (0.0) | 2 (3.8) | >0.999 |
| Congestive heart failure, n (%) | 5 (2.6) | 4 (3.0) | 0 (0.0) | 1 (1.9) | >0.999 |
| Coronary artery disease, n (%) | 6 (3.1) | 6 (4.4) | 0 (0.0) | 0 (0.0) | 0.261 |
| Previous myocardial infarction, n (%) | 5 (2.6) | 5 (3.7) | 0 (0.0) | 0 (0.0) | 0.376 |
| Previous coronary angiography, n (%) | 25 (13.1) | 21 (15.6) | 0 (0.0) | 4 (7.5) | 0.339 |
| Previous percutaneous coronary intervention, n (%) | 5 (2.6) | 5 (3.7) | 0 (0.0) | 0 (0.0) | 0.376 |
| Previous vascular intervention other than coronary, n (%) | 9 (4.7) | 8 (5.9) | 0 (0.0) | 1 (1.9) | 0.524 |
| Chronic kidney disease, n (%) | 11 (5.8) | 10 (7.4) | 0 (0.0) | 1 (1.9) | 0.319 |
| Chronic pulmonary disease, n (%) | 27 (14.1) | 16 (11.9) | 1 (33.3) | 10 (18.9) | 0.169 |
| Chronic inflammatory disease other than rheumatic disease, n (%) | 41 (21.5) | 34 (25.2) | 2 (66.7) | 5 (9.4) | **0.008** |
| **Cardiovascular risk factors** | | | | | |
| Diabetes mellitus, n (%) | 29 (15.2) | 23 (17.0) | 0 (0.0) | 6 (11.3) | 0.622 |
| Arterial hypertension, n (%) | 77 (40.3) | 66 (48.9) | 0 (0.0) | 11 (20.8) | **<0.001** |
| Dyslipidaemia, n (%) | 66 (34.6) | 53 (39.3) | 0 (0.0) | 13 (24.5) | 0.090 |
| Smoking history or active smoker, n (%) | 82 (42.9) | 54 (40.0) | 1 (33.3) | 27 (50.9) | 0.376 |
| Active smoker at time of diagnosis, n (%) | 25 (30.5) | 20 (37.0) | 0 (0.0) | 5 (18.5) | 0.688 |
| Pack years, median (IQR) | 12.00 [5.00, 30.00] | 15.00 [5.00, 30.00] | - | 10.00 [1.00, 30.00] | 0.141 |
| Family history of cardiovascular disease, n (%) | 65 (34.0) | 50 (37.0) | 1 (33.3) | 14 (26.4) | 0.294 |

**Table S2**: Demographics, previous medical history, and cardiovascular risk factors according to vasculitis subgroups.

|  | **Overall**  **(n=191)** | **LVV**  **(n=135)**  **(I)** | **MVV**  **(n=3)**  **(II)** | **SVV**  **(n=53)**  **(III)** | **p-value**  **I vs. II vs. III** |
| --- | --- | --- | --- | --- | --- |
| **Diagnosis of vasculitis** | | | | | |
| Age at diagnosis (years), median (IQR) | 64.00 [48.50, 74.00] | 70.00 [58.50, 77.00] | 44.00 [37.50, 55.50] | 51.00 [38.00, 61.00] | **<0.001** |
| BMI at diagnosis (kg/m^2^), median (IQR) | 24.53 [21.35, 27.10] | 24.39 [21.11, 26.89] | 21.30 [20.32, 21.79] | 25.86 [23.04, 29.38] | **0.015** |
| Number of physician visits until establishment of definite diagnosis (n), median (IQR) | 4.00 [2.00, 9.75] | 3.00 [2.00, 7.00] | 6.00 [4.00, 8.00] | 6.00 [3.00, 10.00] | **0.004** |
| Interval between first physician contact related to vasculitis symptoms and confirmed diagnosis | | | | | |
| <= 1 year, n (%) | 152 (79.6) | 114 (84.4) | 2 (66.7) | 36 (67.9) | **0.035** |
| 1 to 2 years, n (%) | 12 (6.3) | 6 (4.4) | 1 (33.3) | 5 (9.4) | 0.074 |
| 2 to 3 years, n (%) | 8 (4.2) | 5 (3.7) | 0 (0.0) | 3 (5.7) | 0.726 |
| 3 to 4 years, n (%) | 3 (1.6) | 0 (0.0) | 0 (0.0) | 3 (5.7) | **0.042** |
| 4 to 5 years, n (%) | 2 (1.0) | 1 (0.7) | 0 (0.0) | 1 (1.9) | 0.495 |
| > 5 years, n (%) | 10 (5.2) | 7 (5.2) | 0 (0.0) | 3 (5.7) | <0.999 |
| **Medical specialty initially suspecting vasculitis** | | | | | |
| Rheumatology, n (%) | 44 (23.0) | 31 (23.0) | 1 (33.3) | 12 (22.6) | 0.866 |
| Cardiology, n (%) | 16 (8.4) | 12 (8.9) | 0 (0.0) | 4 (7.5) | >0.999 |
| Angiology, n (%) | 17 (8.9) | 17 (12.6) | 0 (0.0) | 0 (0.0) | **0.010** |
| Dermatology, n (%) | 4 (2.1) | 2 (1.5) | 1 (33.3) | 1 (1.9) | 0.067 |
| Ophthalmology, n (%) | 26 (13.6) | 21 (15.6) | 0 (0.0) | 5 (9.4) | 0.585 |
| Neurology, n (%) | 10 (5.2) | 10 (7.4) | 0 (0.0) | 0 (0.0) | 0.126 |
| Orthopedics, n (%) | 6 (3.1) | 6 (4.4) | 0 (0.0) | 0 (0.0) | 0.263 |
| ENT, n (%) | 8 (4.2) | 1 (0.7) | 0 (0.0) | 7 (13.2) | **0.001** |
| Pulmonology, n (%) | 4 (2.1) | 0 (0.0) | 0 (0.0) | 4 (7.5) | **0.007** |
| General medicine, n (%) | 48 (25.1) | 36 (26.7) | 2 (66.7) | 10 (18.9) | 0.128 |
| Emergency medicine, n (%) | 29 (15.2) | 21 (15.6) | 0 (0.0) | 8 (15.1) | >0.999 |
| Other, n (%) | 24 (12.6) | 13 (9.6) | 0 (0.0) | 11 (20.8) | 0.083 |
| **B-symptoms at vasculitis diagnosis** | | | | | |
| Fever, n (%) | 51 (26.7) | 33 (24.4) | 1 (33.3) | 17 (32.1) | 0.433 |
| Arthralgias, n (%) | 102 (53.4) | 68 (50.4) | 1 (33.3) | 33 (62.3) | 0.301 |
| Sicca, n (%) | 57 (29.8) | 40 (29.6) | 1 (33.3) | 16 (30.2) | 0.937 |
| Night sweats, n (%) | 91 (47.6) | 64 (47.4) | 1 (33.3) | 26 (49.1) | 0.853 |
| Myalgia, n (%) | 106 (55.5) | 74 (54.8) | 2 (66.7) | 30 (56.6) | 0.946 |
| Raynaud, n (%) | 27 (14.1) | 18 (13.3) | 1 (33.3) | 8 (15.1) | 0.415 |
| Weight Loss, n (%) | 74 (38.7) | 52 (38.5) | 1 (33.3) | 21 (39.6) | >0.999 |
| Hair Loss, n (%) | 31 (16.2) | 24 (17.8) | 1 (33.3) | 6 (11.3) | 0.310 |
| Bleeding tendency (excluding drug-related), n (%) | 22 (11.5) | 13 (9.6) | 1 (33.3) | 8 (15.1) | 0.177 |

**Table S3**: Diagnosis of vasculitis according to vasculitis subgroups.

|  | **Overall**  **(n=191)** | **LVV**  **(n=135)**  **(I)** | **MVV**  **(n=3)**  **(II)** | **SVV**  **(n=53)**  **(III)** | **p-value**  **I vs. II vs. III** |
| --- | --- | --- | --- | --- | --- |
| **Cardiac symptoms after vasculitis diagnosis** | | | | | |
| Dyspnoea, n (%) | 47 (24.6) | 29 (21.5) | 1 (33.3) | 17 (32.1) | 0.291 |
| Chest pain, n (%) | 34 (17.8) | 17 (12.6) | 1 (33.3) | 16 (30.2) | **0.013** |
| Congestion/oedema, n (%) | 50 (26.2) | 31 (23.0) | 2 (66.7) | 17 (32.1) | 0.082 |
| Palpitations, n (%) | 51 (26.7) | 35 (25.9) | 1 (33.3) | 15 (28.3) | 0.878 |
| Syncope, n (%) | 25 (13.1) | 19 (14.1) | 1 (33.3) | 5 (9.4) | 0.248 |
| **New onset arterial hypertension after vasculitis diagnosis** | | | | | |
| New onset arterial hypertension requiring medical therapy after diagnosis of vasculitis, n (%) | 52 (27.2) | 32 (23.7) | 1 (33.3) | 19 (35.8) | 0.162 |
| **Arrhythmias after vasculitis diagnosis** | | | | | |
| Conduction disorders, n (%) | 12 (6.3) | 8 (5.9) | 0 (0.0) | 4 (7.5) | 0.789 |
| Ventricular arrhythmias, n (%) | 7 (3.7) | 5 (3.7) | 0 (0.0) | 2 (3.8) | >0.999 |
| Supraventricular arrhythmias, n (%) | 22 (11.5) | 17 (12.6) | 0 (0.0) | 5 (9.4) | 0.863 |
| **New structural abnormalities and biopsy findings after vasculitis diagnosis** | | | | | |
| Echocardiography performed, n (%) | 133 (69.6) | 82 (60.7) | 3 (100.0) | 48 (90.6) | **<0.001** |
| LV systolic dysfunction, n (%) | 11 (8.3) | 5 (6.1) | 0 (0.0) | 6 (12.5) | 0.489 |
| Regional wall motion abnormalities, n (%) | 4 (3.0) | 2 (2.4) | 0 (0.0) | 2 (4.2) | 0.613 |
| LV diastolic dysfunction, n (%) | 40 (30.1) | 24 (29.3) | 1 (33.3) | 15 (31.2) | 0.929 |
| RV systolic dysfunction, n (%) | 5 (3.8) | 3 (3.7) | 0 (0.0) | 2 (4.2) | >0.999 |
| Moderate/severe aortic valve regurgitation, n (%) | 4 (3.0) | 4 (4.9) | 0 (0.0) | 0 (0.0) | >0.999 |
| Moderate/severe aortic valve stenosis, n (%) | 7 (5.3) | 3 (3.7) | 1 (33.3) | 3 (6.2) | 0.143 |
| Moderate severe mitral valve regurgitation, n (%) | 12 (9.0) | 9 (11.0) | 1 (33.3) | 2 (4.2) | 0.112 |
| Pericardial effusion, n (%) | 12 (9.0) | 5 (6.1) | 0 (0.0) | 7 (14.6) | 0.356 |
| Tricuspid regurgitation gradient >31mmHg, n (%) | 12 (9.0) | 8 (9.8) | 1 (33.3) | 3 (6.2) | 0.190 |
| Cardiac MRI performed, n (%) | 21 (11.0) | 7 (5.2) | 0 (0.0) | 14 (26.4) | **<0.001** |
| Cardiomyopathy, n (%) | 6 (28.6) | 0 (0.0) | - | 6 (42.9) | 0.051 |
| EMB performed, n (%) | 7 (3.7) | 1 (0.7) | 0 (0.0) | 6 (11.3) | **0.006** |
| Signs of inflammation, n (%) | 3 (42.9) | 0 (0.0) | - | 3 (50.0) | >0.999 |
| Intracardiac thrombus, n (%) | 3 (1.6) | 2 (1.5) | 0 (0.0) | 1 (1.9) | >0.999 |
| **Myocardial ischemia after vasculitis diagnosis** | | | | | |
| Electrocardiographic signs of myocardial ischemia, n (%) | 5 (2.6) | 4 (3.0) | 0 (0.0) | 1 (1.9) | >0.999 |
| Myocardial infarction, n (%) | 4 (2.1) | 4 (3.0) | 0 (0.0) | 0 (0.0) | 0.604 |

**Table S4**: Cardiac symptoms and cardiovascular diagnostic findings after vasculitis diagnosis according to vasculitis subgroups.

|  | **Overall**  **(n=191)** | **LVV**  **(n=135)**  **(I)** | **MVV**  **(n=3)**  **(II)** | **SVV**  **(n=53)**  **(III)** | **p-value**  **I vs. II vs. III** |
| --- | --- | --- | --- | --- | --- |
| **Management of vasculitis** | | | | | |
| Long-term Immunosuppressive medication before vasculitis diagnosis, n (%) | 31 (16.2) | 20 (14.8) | 1 (33.3) | 10 (18.9) | 0.358 |
| Corticosteroids for initial immunomodulatory therapy, n (%) | 189 (99.0) | 134 (99.3) | 3 (100.0) | 52 (98.1) | 0.295 |
| Oral application of corticosteroids, n (%) | 161 (85.2) | 112 (83.6) | 3 (100.0) | 46 (88.5) | 0.797 |
| Intravenous application of corticosteroids, n (%) | 91 (48.1) | 63 (47.0) | 0 (0.0) | 28 (53.8) | 0.460 |
| Adjunctive immunomodulatory therapy, n (%) | 158 (82.7) | 102 (75.6) | 3 (100.0) | 53 (100.0) | **<0.001** |
| Azathioprine, n (%) | 39 (24.7) | 13 (12.7) | 2 (66.7) | 24 (45.3) | **<0.001** |
| Cyclophosphamide, n (%) | 22 (13.9) | 4 (3.9) | 2 (66.7) | 16 (30.2) | **<0.001** |
| IL6-Receptor Antagonist, n (%) | 73 (46.2) | 72 (70.6) | 0 (0.0) | 1 (1.9) | **<0.001** |
| Other adjunctive immunosuppressive therapy, n (%) | 110 (69.6) | 56 (54.9) | 3 (100.0) | 51 (96.2) | **<0.001** |
| Hospital admission for vasculitis treatment, n (%) | 96 (50.3) | 67 (49.6) | 2 (66.7) | 27 (50.9) | 0.853 |
| Major relapse, n (%) | 63 (33.0) | 38 (28.1) | 2 (66.7) | 23 (43.4) | **0.048** |
| **Cardiac procedures** | | | | | |
| Coronary angiography performed, n (%) | 33 (17.3) | 21 (15.6) | 0 (0.0) | 12 (22.6) | 0.426 |
| CAD without indication for revascularization, n (%) | 8 (24.2) | 6 (28.6) | - | 2 (16.7) | 0.419 |
| Percutaneous coronary intervention, n (%) | 6 (18.2) | 5 (23.8) | - | 1 (8.3) | 0.370 |
| Coronary lesions other than atherosclerotic, n (%) | 4 (12.1) | 2 (9.5) | - | 2 (16.7) | >0.999 |
| Coronary artery bypass grafting, n (%) | 2 (6.1) | 1 (4.8) | - | 1 (8.3) | >0.999 |
| Surgical/interventional valvular procedure, n (%) | 8 (4.2) | 7 (5.2) | 0 (0.0) | 1 (1.9) | 0.512 |
| Pacemaker implantation after vasculitis diagnosis, n (%) | 1 (0.5) | 1 (0.7) | 0 (0.0) | 0 (0.0) | >0.999 |
| ICD implantation after vasculitis diagnosis, n (%) | 3 (1.6) | 2 (1.5) | 0 (0.0) | 1 (1.9) | >0.999 |
| **Cardiovascular medication** | | | | | |
| ASA, n (%) | 96 (50.3) | 85 (63.0) | 1 (33.3) | 10 (18.9) | **<0.001** |
| Initiated after vasculitis diagnosis, n (%) | 84 (87.5) | 76 (89.4) | 1 (100.0) | 7 (70.0) | **<0.001** |
| P2Y12 inhibitor, n (%) | 19 (9.9) | 16 (11.9) | 0 (0.0) | 3 (5.7) | 0.479 |
| Initiated after vasculitis diagnosis, n (%) | 14 (73.7) | 11 (68.8) | - | 3 (100.0) | 0.796 |
| Dual antiplatelet therapy, n (%) | 12 (6.3) | 12 (8.9) | 0 (0.0) | 0 (0.0) | **0.041** |
| Initiated after vasculitis diagnosis, n (%) | 8 (66.7) | 8 (66.7) | - | - | - |
| NOAC, n (%) | 39 (20.4) | 31 (23.0) | 0 (0.0) | 8 (15.1) | 0.430 |
| Initiated after vasculitis diagnosis, n (%) | 32 (82.1) | 24 (77.4) | - | 8 (100.0) | 0.883 |
| Vitamin K Antagonist, n (%) | 13 (6.8) | 9 (6.7) | 0 (0.0) | 4 (7.5) | 0.806 |
| Initiated after vasculitis diagnosis, n (%) | 7 (53.8) | 5 (55.6) | - | 2 (50.0) | >0.999 |
| Betablocker, n (%) | 80 (41.9) | 64 (47.4) | 1 (33.3) | 15 (28.3) | **0.046** |
| Initiated after vasculitis diagnosis, n (%) | 61 (76.2) | 48 (75.0) | 1 (100.0) | 12 (80.0) | 0.126 |
| Diuretics, n (%) | 64 (33.5) | 48 (35.6) | 0 (0.0) | 16 (30.2) | 0.541 |
| Initiated after vasculitis diagnosis, n (%) | 53 (82.8) | 40 (83.3) | - | 13 (81.2) | 0.676 |
| Aldosterone Antagonist, n (%) | 6 (3.1) | 5 (3.7) | 0 (0.0) | 1 (1.9) | >0.999 |
| Initiated after vasculitis diagnosis, n (%) | 4 (66.7) | 3 (60.0) | - | 1 (100.0) | >0.999 |
| ARNI, n (%) | 4 (2.1) | 2 (1.5) | 0 (0.0) | 2 (3.8) | 0.358 |
| Initiated after vasculitis diagnosis, n (%) | 4 (100.0) | 2 (100.0) | - | 2 (100.0) | 0.350 |
| SGLT2-inhibitor, n (%) | 17 (8.9) | 11 (8.1) | 0 (0.0) | 6 (11.3) | 0.678 |
| Initiated after vasculitis diagnosis, n (%) | 14 (82.4) | 9 (81.8) | - | 5 (83.3) | 0.613 |
| ACE-inhibitor/AT1-receptor antagonist, n (%) | 115 (60.2) | 85 (63.0) | 1 (33.3) | 29 (54.7) | 0.323 |
| Initiated after vasculitis diagnosis, n (%) | 88 (76.5) | 65 (76.5) | 1 (100.0) | 22 (75.9) | 0.526 |
| Statin, n (%) | 119 (62.3) | 93 (68.9) | 1 (33.3) | 25 (47.2) | **0.008** |
| Initiated after vasculitis diagnosis, n (%) | 108 (90.8) | 84 (90.3) | 1 (100.0) | 23 (92.0) | **0.021** |
| Calcium antagonist, n (%) | 50 (26.2) | 39 (28.9) | 0 (0.0) | 11 (20.8) | 0.427 |
| Initiated after vasculitis diagnosis, n (%) | 40 (80.0) | 31 (79.5) | - | 9 (81.8) | 0.653 |
| Antiarrhythmic medication (other than betablocker), n (%) | 28 (14.7) | 23 (17.0) | 0 (0.0) | 5 (9.4) | 0.424 |
| Initiated after vasculitis diagnosis, n (%) | 21 (75.0) | 17 (73.9) | - | 4 (80.0) | 0.564 |

**Table S5**: Management of vasculitis and cardiac disease according to vasculitis subgroups.

|  | **GCA (n=109)**  **(I)** | **TAK (n=26)**  **(II)** | **p-value**  **(I vs. II)** | **GPA (n=38)**  **(III)** | **EGPA (n=15)**  **(IV)** | **p-value (III vs. IV)** |
| --- | --- | --- | --- | --- | --- | --- |
| **Demographics** | | | | | | |
| Age at last follow-up (years), median (IQR) | 77.00 [71.00, 83.00] | 43.00 [30.25, 49.75] | **<0.001** | 60.00 [48.00, 72.00] | 61.00 [48.50, 68.00] | 0.746 |
| Sex (male), n (%) | 37 (33.9) | 2 (7.7) | **0.007** | 21 (55.3) | 7 (46.7) | 0.761 |
| **Previous medical history** | | | | | | |
| Cardiomyopathy, n (%) | 3 (2.8) | 2 (7.7) | 0.246 | 1 (2.6) | 1 (6.7) | 0.490 |
| Congestive heart failure, n (%) | 4 (3.7) | 0 (0.0) | >0.999 | 0 (0.0) | 1 (6.7) | 0.283 |
| Coronary artery disease, n (%) | 6 (5.5) | 0 (0.0) | 0.596 | 0 (0.0) | 0 (0.0) | - |
| Previous myocardial infarction, n (%) | 5 (4.6) | 0 (0.0) | 0.583 | 0 (0.0) | 0 (0.0) | - |
| Previous coronary angiography, n (%) | 20 (18.3) | 1 (3.8) | 0.076 | 2 (5.3) | 2 (13.3) | 0.568 |
| Previous percutaneous coronary intervention, n (%) | 5 (4.6) | 0 (0.0) | 0.583 | 0 (0.0) | 0 (0.0) | - |
| Previous vascular intervention other than coronary, n (%) | 6 (5.5) | 2 (7.7) | 0.650 | 1 (2.6) | 0 (0.0) | >0.999 |
| Chronic kidney disease, n (%) | 10 (9.2) | 0 (0.0) | 0.208 | 1 (2.6) | 0 (0.0) | >0.999 |
| Chronic pulmonary disease, n (%) | 16 (14.7) | 0 (0.0) | **0.041** | 0 (0.0) | 10 (66.7) | **<0.001** |
| Chronic inflammatory disease other than rheumatic disease, n (%) | 25 (22.9) | 9 (34.6) | 0.220 | 2 (5.3) | 3 (20.0) | 0.131 |
| **Cardiovascular risk factors** | | | | | | |
| Diabetes mellitus, n (%) | 21 (19.3) | 2 (7.7) | 0.245 | 6 (15.8) | 0 (0.0) | 0.167 |
| Arterial hypertension, n (%) | 61 (56.0) | 5 (19.2) | **0.001** | 8 (21.1) | 3 (20.0) | >0.999 |
| Dyslipidaemia, n (%) | 50 (45.9) | 3 (11.5) | **0.001** | 11 (28.9) | 2 (13.3) | 0.305 |
| Smoking history or active smoker, n (%) | 47 (43.1) | 7 (26.9) | 0.181 | 19 (50.0) | 8 (53.3) | >0.999 |
| Active smoker at time of diagnosis, n (%) | 16 (14.7) | 4 (15.4) | >0.999 | 4 (10.5) | 1 (6.7) | >0.999 |
| Pack years, median (IQR) | 15.00 [5.50, 30.00] | 10.00 [3.50, 13.75] | 0.106 | 7.50 [1.00, 27.50] | 12.50 [8.62, 45.00] | 0.072 |
| Family history of cardiovascular disease, n (%) | 43 (39.4) | 7 (26.9) | 0.362 | 11 (28.9) | 3 (20.0) | 0.732 |

**Table S6**: Intragroup comparisons among patients with large-vessel and small-vessel vasculitis: Demographics, previous medical history, and cardiovascular risk factors.

|  | **GCA (n=109)**  **(I)** | **TAK (n=26)**  **(II)** | **p-value (I vs. II)** | **GPA (n=38)**  **(III)** | **EGPA (n=15)**  **(IV)** | **p-value (III vs. IV)** |
| --- | --- | --- | --- | --- | --- | --- |
| **Diagnosis of vasculitis** | | | | | | |
| Age at diagnosis (years), median (IQR) | 72.00 [66.00, 79.00] | 30.50 [22.25, 40.00] | **<0.001** | 51.00 [38.00, 59.25] | 53.00 [44.50, 64.50] | 0.374 |
| BMI at diagnosis (kg/m^2^), median (IQR) | 24.65 [21.90, 26.98] | 21.05 [19.87, 26.35] | **0.036** | 26.03 [23.48, 28.85] | 25.86 [21.98, 30.72] | 0.737 |
| Number of physician visits until establishment of definite diagnosis (n), median (IQR) | 3.00 [2.00, 5.00] | 10.00 [5.00, 14.25] | **<0.001** | 7.00 [3.00, 10.00] | 6.00 [3.00, 9.00] | 0.613 |
| Interval between first physician contact related to vasculitis symptoms and confirmed diagnosis | | | | | | |
| <= 1 year, n (%) | 98 (89.9) | 16 (61.5) | **0.007** | 27 (71.1) | 9 (60.0) | 0.325 |
| 1 to 2 years, n (%) | 5 (4.6) | 1 (3.8) | >0.999 | 3 (7.9) | 2 (13.3) | 0.624 |
| 2 to 3 years, n (%) | 3 (2.8) | 2 (7.7) | 0.221 | 3 (7.9) | 0 (0.0) | 0.546 |
| 3 to 4 years, n (%) | 0 (0.0) | 0 (0.0) | *-* | 2 (5.3) | 1 (6.7) | >0.999 |
| 4 to 5 years, n (%) | 1 (0.9) | 0 (0.0) | >0.999 | 1 (2.6) | 0 (0.0) | >0.999 |
| > 5 years, n (%) | 2 (1.8) | 5 (19.2) | **0.002** | 0 (0.0) | 3 (20.0) | **0.022** |
| **Medical specialty initially suspecting vasculitis** | | | | | | |
| Rheumatology, n (%) | 21 (19.3) | 10 (38.5) | 0.066 | 7 (18.4) | 5 (33.3) | 0.293 |
| Cardiology, n (%) | 7 (6.4) | 5 (19.2) | 0.054 | 0 (0.0) | 4 (26.7) | **0.005** |
| Angiology, n (%) | 12 (11.0) | 5 (19.2) | 0.321 | 0 (0.0) | 0 (0.0) | - |
| Dermatology, n (%) | 2 (1.8) | 0 (0.0) | >0.999 | 1 (2.6) | 0 (0.0) | >0.999 |
| Ophthalmology, n (%) | 21 (19.3) | 0 (0.0) | **0.013** | 4 (10.5) | 1 (6.7) | >0.999 |
| Neurology, n (%) | 10 (9.2) | 0 (0.0) | 0.208 | 0 (0.0) | 0 (0.0) | - |
| Orthopedics, n (%) | 4 (3.7) | 2 (7.7) | 0.326 | 0 (0.0) | 0 (0.0) | *-* |
| ENT, n (%) | 1 (0.9) | 0 (0.0) | >0.999 | 6 (15.8) | 1 (6.7) | 0.658 |
| Pulmonology, n (%) | 0 (0.0) | 0 (0.0) | - | 1 (2.6) | 3 (20.0) | 0.067 |
| General medicine, n (%) | 29 (26.6) | 7 (26.9) | >0.999 | 10 (26.3) | 0 (0.0) | **0.046** |
| Emergency medicine, n (%) | 21 (19.3) | 0 (0.0) | **0.013** | 3 (7.9) | 5 (33.3) | **0.036** |
| Other, n (%) | 10 (9.2) | 3 (11.5) | 0.715 | 10 (26.3) | 1 (6.7) | 0.145 |
| **B-symptoms at vasculitis diagnosis** | | | | | | |
| Fever, n (%) | 28 (25.7) | 5 (19.2) | 0.618 | 14 (36.8) | 3 (20.0) | 0.328 |
| Arthralgias, n (%) | 58 (53.2) | 10 (38.5) | 0.191 | 24 (63.2) | 9 (60.0) | >0.999 |
| Sicca, n (%) | 37 (33.9) | 3 (11.5) | **0.048** | 11 (28.9) | 5 (33.3) | >0.999 |
| Night sweats, n (%) | 57 (52.3) | 7 (26.9) | **0.028** | 21 (55.3) | 5 (33.3) | 0.132 |
| Myalgia, n (%) | 62 (56.9) | 12 (46.2) | 0.505 | 19 (50.0) | 11 (73.3) | 0.217 |
| Raynaud, n (%) | 14 (12.8) | 4 (15.4) | 0.745 | 6 (15.8) | 2 (13.3) | >0.999 |
| Weight Loss, n (%) | 43 (39.4) | 9 (34.6) | 0.823 | 15 (39.5) | 6 (40.0) | >0.999 |
| Hair Loss, n (%) | 19 (17.4) | 5 (19.2) | 0.783 | 3 (7.9) | 3 (20.0) | 0.343 |
| Bleeding tendency (excluding drug-related), n (%) | 12 (11.0) | 1 (3.8) | 0.461 | 7 (18.4) | 1 (6.7) | 0.412 |

**Table S7**: Intragroup comparisons among patients with large-vessel and small-vessel vasculitis: Diagnosis of vasculitis.

|  | **GCA (n=109)**  **(I)** | **TAK (n=26)**  **(II)** | **p-value**  **(I vs. II)** | **GPA (n=38)**  **(III)** | **EGPA (n=15)**  **(IV)** | **p-value (III vs. IV)** |
| --- | --- | --- | --- | --- | --- | --- |
| **Cardiac symptoms after vasculitis diagnosis** | | | | | | |
| Dyspnoea, n (%) | 24 (22.0) | 5 (19.2) | 0.797 | 10 (26.3) | 7 (46.7) | 0.197 |
| Chest pain, n (%) | 13 (11.9) | 4 (15.4) | 0.742 | 9 (23.7) | 7 (46.7) | 0.182 |
| Congestion/oedema, n (%) | 29 (26.6) | 2 (7.7) | **0.040** | 9 (23.7) | 8 (53.3) | 0.056 |
| Palpitations, n (%) | 26 (23.9) | 9 (34.6) | 0.319 | 8 (21.1) | 7 (46.7) | 0.091 |
| Syncope, n (%) | 18 (16.5) | 1 (3.8) | 0.122 | 3 (7.9) | 2 (13.3) | 0.614 |
| **New onset arterial hypertension after vasculitis diagnosis** | | | | | | |
| New onset arterial hypertension requiring medical therapy after diagnosis of vasculitis, n (%) | 23 (21.1) | 9 (34.6) | 0.198 | 15 (39.5) | 4 (26.7) | 0.528 |
| **Arrhythmias after vasculitis diagnosis** | | | | | | |
| Conduction disorders, n (%) | 6 (5.5) | 2 (7.7) | 0.644 | 2 (5.3) | 2 (13.3) | 0.569 |
| Ventricular arrhythmias, n (%) | 4 (3.7) | 1 (3.8) | >0.999 | 0 (0.0) | 2 (13.3) | 0.079 |
| Supraventricular arrhythmias, n (%) | 16 (14.7) | 1 (3.8) | 0.195 | 3 (7.9) | 2 (13.3) | 0.619 |
| **New structural abnormalities and biopsy findings after vasculitis diagnosis** | | | | | | |
| Echocardiography performed, n (%) | 65 (59.6) | 17 (65.4) | 0.659 | 35 (92.1) | 13 (86.7) | 0.614 |
| LV systolic dysfunction, n (%) | 5 (7.7) | 0 (0.0) | 0.577 | 3 (8.6) | 3 (23.1) | 0.323 |
| Regional wall motion abnormalities, n (%) | 1 (1.5) | 1 (5.9) | 0.478 | 1 (2.9) | 1 (7.7) | 0.181 |
| LV diastolic dysfunction, n (%) | 18 (27.7) | 6 (35.3) | 0.534 | 11 (31.4) | 4 (30.8) | >0.999 |
| RV systolic dysfunction, n (%) | 3 (4.6) | 0 (0.0) | >0.999 | 2 (5.7) | 0 (0.0) | >0.999 |
| Moderate/severe aortic valve regurgitation, n (%) | 3 (4.6) | 1 (5.9) | >0.999 | 0 (0.0) | 0 (0.0) | - |
| Moderate/severe aortic valve stenosis, n (%) | 3 (4.6) | 0 (0.0) | >0.999 | 3 (8.6) | 0 (0.0) | 0.553 |
| Moderate severe mitral valve regurgitation, n (%) | 7 (10.8) | 2 (11.8) | >0.999 | 1 (2.9) | 1 (7.7) | 0.473 |
| Pericardial effusion, n (%) | 3 (4.6) | 2 (11.8) | 0.254 | 3 (8.6) | 4 (30.8) | 0.080 |
| Tricuspid regurgitation gradient >31mmHg, n (%) | 7 (10.8) | 1 (5.9) | >0.999 | 1 (2.9) | 2 (15.4) | 0.181 |
| Cardiac MRI performed, n (%) | 5 (4.6) | 2 (7.7) | 0.622 | 4 (10.5) | 10 (66.7) | **<0.001** |
| Cardiomyopathy, n (%) | 0 (0.0) | 0 (0.0) | - | 1 (25.0) | 5 (50.0) | >0.999 |
| EMB performed, n (%) | 1 (0.9) | 0 (0.0) | >0.999 | 0 (0.0) | 6 (40.0) | **<0.001** |
| Signs of inflammation, n (%) | 0 (0.0) | - | - | - | 3 (50.0) | >0.999 |
| Intracardiac thrombus, n (%) | 2 (1.8) | 0 (0.0) | >0.999 | 0 (0.0) | 1 (6.7) | 0.283 |
| **Myocardial ischemia after vasculitis diagnosis** | | | | | | |
| Electrocardiographic signs of myocardial ischemia, n (%) | 3 (2.8) | 1 (3.8) | 0.573 | 0 (0.0) | 1 (6.7) | 0.351 |
| Myocardial infarction, n (%) | 4 (3.7) | 0 (0.0) | >0.999 | 0 (0.0) | 0 (0.0) | - |

**Table S8**: Intragroup comparisons among patients with large-vessel and small-vessel vasculitis: Cardiac symptoms and cardiovascular diagnostic findings after vasculitis diagnosis.

|  | **GCA (n=109)**  **(I)** | **TAK (n=26)**  **(II)** | **p-value**  **(I vs. II)** | **GPA (n=38)**  **(III)** | **EGPA (n=15)**  **(IV)** | **p-value (III vs. IV)** |
| --- | --- | --- | --- | --- | --- | --- |
| **Management of vasculitis** | | | | | | |
| Long-term Immunosuppressive medication before vasculitis diagnosis, n (%) | 15 (13.8) | 5 (19.2) | 0.534 | 4 (10.5) | 6 (40.0) | **0.024** |
| Corticosteroids for initial immunomodulatory therapy, n (%) | 109 (100.0) | 25 (96.2) | - | 37 (97.4) | 15 (100.0) | >0.999 |
| Oral application of corticosteroids, n (%) | 87 (79.8) | 25 (100.0) | **0.047** | 34 (91.9) | 12 (80.0) | 0.389 |
| Intravenous application of corticosteroids, n (%) | 57 (52.3) | 6 (24.0) | **0.005** | 19 (51.4) | 9 (60.0) | 0.533 |
| Adjunctive immunomodulatory therapy, n (%) | 77 (70.6) | 25 (96.2) | **0.005** | 38 (100.0) | 15 (100.0) | *-* |
| Azathioprine, n (%) | 7 (9.1) | 6 (24.0) | **0.016** | 18 (47.4) | 6 (40.0) | 0.760 |
| Cyclophosphamide, n (%) | 2 (2.6) | 2 (8.0) | 0.169 | 11 (28.9) | 5 (33.3) | 0.751 |
| IL6-Receptor Antagonist, n (%) | 61 (79.2) | 11 (44.0) | 0.262 | 1 (2.6) | 0 (0.0) | >0.999 |
| Other adjunctive immunosuppressive therapy, n (%) | 31 (40.3) | 25 (100.0) | **<0.001** | 37 (97.4) | 14 (93.3) | 0.490 |
| Hospital admission for vasculitis treatment, n (%) | 54 (49.5) | 13 (50.0) | >0.999 | 16 (42.1) | 11 (73.3) | 0.073 |
| Major relapse, n (%) | 29 (26.6) | 9 (34.6) | 0.469 | 19 (50.0) | 4 (26.7) | 0.140 |
| **Cardiac procedures** | | | | | | |
| Coronary angiography performed, n (%) | 19 (17.4) | 2 (7.7) | 0.365 | 5 (13.2) | 7 (46.7) | **0.024** |
| CAD without indication for revascularization, n (%) | 6 (31.6) | 0 (0.0) | 0.529 | 1 (20.0) | 1 (14.3) | >0.999 |
| Percutaneous coronary intervention, n (%) | 4 (21.1) | 1 (50.0) | 0.447 | 1 (20.0) | 0 (0.0) | 0.417 |
| Coronary lesions other than atherosclerotic, n (%) | 2 (10.5) | 0 (0.0) | >0.999 | 2 (40.0) | 0 (0.0) | 0.152 |
| Coronary artery bypass grafting, n (%) | 1 (5.3) | 0 (0.0) | >0.999 | 1 (20.0) | 0 (0.0) | 0.417 |
| Surgical/interventional valvular procedure, n (%) | 6 (5.5) | 1 (3.8) | >0.999 | 1 (2.6) | 0 (0.0) | >0.999 |
| Pacemaker implantation after vasculitis diagnosis, n (%) | 1 (0.9) | 0 (0.0) | >0.999 | 0 (0.0) | 0 (0.0) | - |
| ICD implantation after vasculitis diagnosis, n (%) | 2 (1.8) | 0 (0.0) | >0.999 | 0 (0.0) | 1 (6.7) | - |
| **Cardiovascular medication** | | | | | | |
| ASA, n (%) | 66 (60.6) | 19 (73.1) | 0.266 | 6 (15.8) | 4 (26.7) | 0.442 |
| Initiated after vasculitis diagnosis, n (%) | 57 (86.4) | 19 (100.0) | 0.082 | 5 (83.3) | 2 (50.0) | >0.999 |
| P2Y12 inhibitor, n (%) | 11 (10.1) | 5 (19.2) | 0.193 | 2 (5.3) | 1 (6.7) | >0.999 |
| Initiated after vasculitis diagnosis, n (%) | 6 (54.4) | 5 (100.0) | **0.039** | 2 (100.0) | 1 (100.0) | >0.999 |
| Dual antiplatelet therapy, n (%) | 8 (7.3) | 4 (15.4) | 0.245 | 0 (0.0) | 0 (0.0) | - |
| Initiated after vasculitis diagnosis, n (%) | 4 (50.0) | 4 (100.0) | **0.046** | - | - | - |
| NOAC, n (%) | 30 (27.5) | 1 (3.8) | **0.009** | 5 (13.2) | 3 (20.0) | 0.673 |
| Initiated after vasculitis diagnosis, n (%) | 23 (76.7) | 1 (100.0) | **0.044** | 5 (100.0) | 3 (100.0) | 0.679 |
| Vitamin K Antagonist, n (%) | 8 (7.3) | 1 (3.8) | >0.999 | 3 (7.9) | 1 (6.7) | >0.999 |
| Initiated after vasculitis diagnosis, n (%) | 4 (50.0) | 1 (100.0) | >0.999 | 2 (66.7) | 0 (0.0) | >0.999 |
| Betablocker, n (%) | 57 (52.3) | 7 (26.9) | **0.028** | 6 (15.8) | 9 (60.0) | **0.005** |
| Initiated after vasculitis diagnosis, n (%) | 42 (73.7) | 6 (85.7) | 0.115 | 4 (66.7) | 8 (88.9) | **0.003** |
| Diuretics, n (%) | 41 (37.6) | 7 (26.9) | 0.367 | 9 (23.7) | 7 (46.7) | 0.182 |
| Initiated after vasculitis diagnosis, n (%) | 34 (82.9) | 6 (85.7) | 0.477 | 8 (88.9) | 5 (71.4) | 0.493 |
| Aldosterone Antagonist, n (%) | 3 (2.8) | 2 (7.7) | 0.246 | 0 (0.0) | 1 (6.7) | 0.283 |
| Initiated after vasculitis diagnosis, n (%) | 2 (66.7) | 1 (50.0) | 0.476 | - | 1 (100.0) | 0.300 |
| ARNI, n (%) | 2 (1.8) | 0 (0.0) | >0.999 | 0 (0.0) | 2 (13.3) | 0.076 |
| Initiated after vasculitis diagnosis, n (%) | 2 (100.0) | - | >0.999 | - | 2 (100.0) | 0.086 |
| SGLT2-inhibitor, n (%) | 10 (9.2) | 1 (3.8) | 0.690 | 2 (5.3) | 4 (26.7) | **0.047** |
| Initiated after vasculitis diagnosis, n (%) | 8 (80.0) | 1 (100.0) | 0.687 | 2 (100.0) | 3 (75.0) | 0.152 |
| ACE-inhibitor/AT1-receptor antagonist, n (%) | 74 (67.9) | 11 (42.3) | **0.023** | 22 (57.9) | 7 (46.7) | 0.547 |
| Initiated after vasculitis diagnosis, n (%) | 56 (75.7) | 9 (81.8) | 0.258 | 16 (72.7) | 6 (85.7) | >0.999 |
| Statin, n (%) | 86 (78.9) | 7 (26.9) | **<0.001** | 18 (47.4) | 7 (46.7) | >0.999 |
| Initiated after vasculitis diagnosis, n (%) | 77 (89.5) | 7 (100.0  ) | **<0.001** | 16 (88.9) | 7 (100.0  ) | >0.999 |
| Calcium antagonist, n (%) | 34 (31.2) | 5 (19.2) | 0.335 | 8 (21.1) | 3 (20.0) | >0.999 |
| Initiated after vasculitis diagnosis, n (%) | 26 (76.5) | 5 (100.0) | 0.795 | 8 (100.0) | 1 (33.3) | 0.251 |
| Antiarrhythmic medication (other than betablocker), n (%) | 22 (20.2) | 1 (3.8) | **0.047** | 3 (7.9) | 2 (13.3) | 0.614 |
| Initiated after vasculitis diagnosis, n (%) | 17 (77.3) | 0 (0.0) | **0.042** | 2 (66.7) | 2 (100.0) | 0.574 |

**Table S9**: Intragroup comparisons among patients with large-vessel and small-vessel vasculitis: Management of vasculiti and cardiac disease.
